# Phagocytosis-dependent activation of a TLR9–BTK–calcineurin–NFAT pathway co-ordinates innate immunity to *Aspergillus fumigatus*

**DOI:** 10.15252/emmm.201404556

**Published:** 2015-01-30

**Authors:** Susanne Herbst, Anand Shah, Maria Mazon Moya, Vanessa Marzola, Barbara Jensen, Anna Reed, Mark A Birrell, Shinobu Saijo, Serge Mostowy, Sunil Shaunak, Darius Armstrong-James

**Affiliations:** 1 Department of Infectious Diseases and Immunity, Imperial College LondonLondon, UK; 2 MRC Centre for Molecular Bacteriology and Infection, Imperial College LondonLondon, UK; 3 Lung Transplant Unit, Royal Brompton and Harefield HospitalLondon, UK; 4 National Heart and Lung Institute, Imperial College LondonLondon, UK; 5 Medical Mycology Research Centre, Chiba UniversityChiba, Japan

**Keywords:** aspergillus, calcineurin, phagocytosis, TLR9, transplant

## Abstract

Transplant recipients on calcineurin inhibitors are at high risk of invasive fungal infection. Understanding how calcineurin inhibitors impair fungal immunity is a key priority for defining risk of infection. Here, we show that the calcineurin inhibitor tacrolimus impairs clearance of the major mould pathogen *Aspergillus fumigatus* from the airway, by inhibiting macrophage inflammatory responses. This leads to defective early neutrophil recruitment and fungal clearance. We confirm these findings in zebrafish, showing an evolutionarily conserved role for calcineurin signalling in neutrophil recruitment during inflammation. We find that calcineurin–NFAT activation is phagocytosis dependent and collaborates with NF-κB for TNF-α production. For yeast zymosan particles, activation of macrophage calcineurin–NFAT occurs via the phagocytic Dectin-1–spleen tyrosine kinase pathway, but for *A. fumigatus*, activation occurs via a phagosomal TLR9-dependent and Bruton's tyrosine kinase-dependent signalling pathway that is independent of MyD88. We confirm the collaboration between NFAT and NF-κB for TNF-α production in primary alveolar macrophages. These observations identify inhibition of a newly discovered macrophage TLR9–BTK–calcineurin–NFAT signalling pathway as a key immune defect that leads to organ transplant-related invasive aspergillosis.

## Introduction

Fungal pathogens have become a major threat to human health, due to an increasing number of invasive infections, and as a serious risk to food security and biosystems (Brown *et al*, 2012; Fisher *et al*, 2012; Armstrong-James *et al*, 2014). In particular, invasive fungal infections are life threatening in immunocompromised patients, even with optimal medical therapy (Kontoyiannis *et al*, 2010; Pappas *et al*, 2010). Whilst neutropaenia is a key risk factor for pulmonary infection with the major mould pathogen *Aspergillus fumigatus* (AF) in patients receiving chemotherapy or stem cell transplantation, solid organ transplant recipients and patients with graft-versus-host disease represent an enlarging population of at-risk individuals in whom susceptibility to invasive infection is more complex and unpredictable (Baddley *et al*, 2010). The relative contribution of innate and adaptive immune responses to fungi has become a key goal for understanding why certain patients develop invasive disease and for the rational design of targeted immunotherapy and vaccination against fungi in at-risk patient groups (Armstrong-James & Harrison, 2012).

Calcineurin inhibitors are the most commonly used class of drugs for the prevention of solid organ transplant rejection and are now routinely used for immunosuppressive therapy for a range of other immunological conditions such as post-haematopoetic stem cell transplant graft-versus-host disease, rheumatoid arthritis and atopic dermatitis (Lee *et al*, 2010; Penninga *et al*, 2010; Svensson *et al*, 2011). The first clinically licensed calcineurin inhibitor, ciclosporin, and the newer and now more commonly used calcineurin inhibitor, tacrolimus (FK506), are both thought to act through inhibition of T-cell calcium-dependent calcineurin–nuclear factor of T cells (NFAT) activation (Fung, 2004). This leads to inhibition of T-cell cytokine responses, and this reduces the risk of T-cell-mediated graft rejection. However, a number of studies have demonstrated a wider role for calcineurin–NFAT signalling in calcium-dependent cellular responses across a range of different tissues and cell types (Kao *et al*, 2009; Zeini *et al*, 2009; Zaslavsky *et al*, 2013). In particular, aberrant calcineurin–NFAT signalling has been shown to play a role in the pathogenesis of both cardiac and neurological syndromes (Abdul *et al*, 2009; Panther *et al*, 2009).

Emerging data have also demonstrated a role for calcineurin–NFAT signalling in innate immune responses (Fric *et al*, 2012). Initial studies suggested that calcineurin–NFAT signalling is required for normal development of lymphocyte populations, but not myeloid lineages. However, it has recently been shown that calcineurin–NFAT signalling plays a major role in osteoclast differentiation, mast cell degranulation and LPS responses via endocytosis of CD14 (Yarilina *et al*, 2011; Zanoni *et al*, 2012; Yissachar *et al*, 2013). Furthermore, our group and others have shown a key requirement for competent calcineurin-dependent innate immune responses in murine models of solid organ transplant infection (Greenblatt *et al*, 2010; Herbst *et al*, 2013; Tourneur *et al*, 2013). Further studies in man indicate that renal transplant recipients at risk of invasive fungal infections also have profoundly defective TNF-α expression (Armstrong-James *et al*, 2012). These observations have led to a major paradigm shift in our understanding of susceptibility to fungal infections in solid organ transplant patients and suggest that defects in innate rather than adaptive immune responses may be the primary driver for invasive fungal infections following transplant immunosuppression.

A better understanding of the underlying mechanisms activating innate calcineurin–NFAT signalling in response to pathogens and a clearer definition of NFAT-dependent innate immune response are now required. For *Candida albicans*, initial studies suggest that the C-type lectin receptor Dectin-1 mediates calcineurin–NFAT activation resulting in competent IL-10 responses and optimal killing by neutrophils (Goodridge *et al*, 2007; Greenblatt *et al*, 2010). However, the mechanistic basis for calcineurin-dependent fungal killing remains to be elucidated. Whilst Dectin-1 has clearly beenshown to be a dominant receptor required for immunity to *C. albicans*, innate immune responses to AF appear more complexand involve both Dectin-1 and Dectin-2, as well as Toll-like receptors, complement and opsonins (Richardson *et al*, 1991; Madan *et al*, 1997; Hohl *et al*, 2005; Luther *et al*, 2007; Werner *et al*, 2009; Moalli *et al*, 2010; Hasenberg *et al*, 2011; Carrion Sde*et al*, 2013). The cytoplasmic tail of Dectin-1 contains a hemi-ITAM motif that is phosphorylated upon Dectin-1 ligation. ITAM phosphorylation activates signalling through the Syk (spleen tyrosine kinase)–CARD9 (caspase recruitment domain containing protein) pathway and the RAF pathway, leading to NF-κB activation and cytokine expression (Rogers *et al*, 2005; Gross *et al*, 2006; Ifrim *et al*, 2013). In addition, Syk (Carrion Sde *et al*, 2013) signalling activates the NLRP3 inflammasome (Gross *et al*, 2009). However, the key intracellular signalling pathways that lead to fungal calcineurin–NFAT activation, and how they integrate with other known inflammatory pathways, have not been characterized in detail.

In this study, we show that calcineurin inhibition leads to defective early chemokine responses to AF in the airway, leading to impaired neutrophil recruitment and fungal killing *in vivo*. We confirm the neutrophil recruitment defect in a zebrafish model of invasive aspergillosis and demonstrate a general role for calcineurin signalling in leucocyte trafficking using a tail-cut trauma model. Our *in vitro* studies demonstrate that the calcineurin–NFAT pathway co-operates with the NF-κB pathway to co-ordinate TNF-α responses in macrophages. We also show a key requirement for phagocytosis in the activation of calcineurin–NFAT signalling and demonstrate that calcineurin activation in response to AF occurs through a MyD88-independent pathway that is TLR9 and Bruton's tyrosine kinase (BTK) dependent. Complementary clinical studies in human lung transplant recipient alveolar macrophages further confirm the collaboration between calcineurin–NFAT and NF-κB pathways for AF-dependent TNF-α responses. These studies therefore define the TLR9–BTK–calcineurin–NFAT pathway as an innate intracellular recognition system that is critically impaired in transplant-related pulmonary aspergillosis.

## Results

### The calcineurin inhibitor FK506 impairs airway innate responses in pulmonary aspergillosis through a steroid-independent mechanism

**Figure 1:**
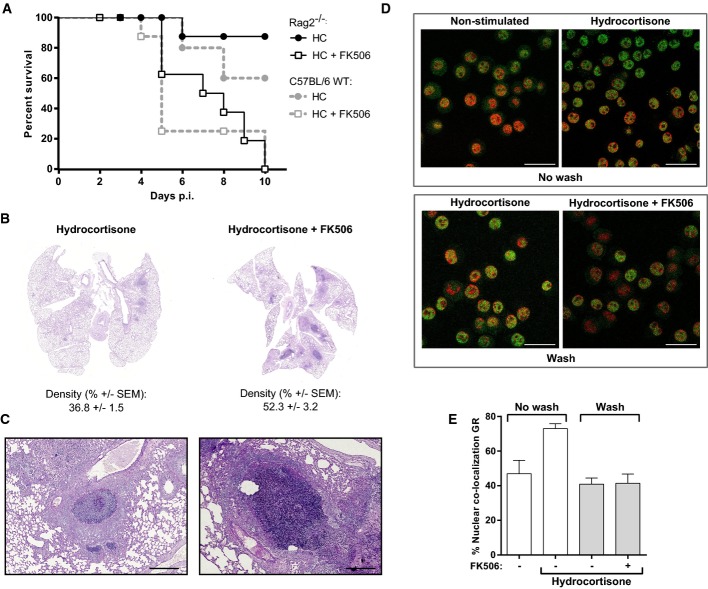
The calcineurin inhibitor FK506 impairs innate immunity to *Aspergillus fumigatus*
A C57BL/6 WT or Rag2^−/−^ mice were immunosuppressed with hydrocortisone +/− FK506 and infected with 10^5^ AF CEA10 conidia intranasally. Mortality was assessed using a 20% weight loss endpoint. Mortality was significantly increased in FK506 immunosuppressed mice reaching 100% by day 10 p.i. (*P *=* *0.003 for HC versus HC+FK506 in Rag2^−/−^ using log-rank test, *n *=* *10 per group).B, C PAS-stained lung sections of Rag2^−/−^ mice were scanned and inflammation quantified by pixel intensity threshold analysis in ImageJ. FK506-treated animals had increased inflammation at day 2 p.i. compared to control. *P *=* *0.0126 (using Student's *t*-test), *n *=* *3-5 per group. (C) Close-up of an infectious focus showing increased inflammation and fungal growth in the FK506-treated group. Scale bar: 400 μm.D, E Murine macrophages (J7741.A) were pre-treated with hydrocortisone, and the effects of FK506 on nuclear shuttling of the glucocorticoid receptor were assessed by confocal immunofluorescence microscopy. The nuclei are indicated in red, and the GR is indicated in green. Scale bar: 50 μm. FK506 did not cause increased translocation of the glucocorticoid receptor. Data represent mean + SEM calculated from 10 fields of vision. One out of 2 experiments is shown.

Previous studies in a hydrocortisone-based model of invasive pulmonary aspergillosis (IPA) indicated that FK506 impaired the early inflammatory response to AF in the lung (Herbst *et al*, 2013). We therefore assessed the effects of FK506 on outcome from pulmonary aspergillosis in RAG^−/−^ mice that lack competent adaptive immune responses. Addition of FK506 to hydrocortisone (HC) immunosuppression protocols in RAG^−/−^ mice led to incremental mortality from IPA, consistent with an effect of FK506 on innate immune responses to AF (Fig1A). Histopathological examination of lung sections demonstrated increased inflammation and fungal invasion in the FK506-treated animals (Fig1B and C).

Earlier studies have suggested that high doses of FK506 may increase nuclear retention of the glucocorticoid receptor (GR) during steroid therapy, via the FK506-binding protein 51 (FKBP51) (Zhang *et al*, 2008). We considered that this may be the primary mechanism by which FK506 impairs innate antifungal responses in transplant recipients. To test this, we investigated the effects of FK506 on hydrocortisone-induced GR translocation in murine macrophages. Macrophages were treated with hydrocortisone to induce GR translocation. After 30 min, cells were washed three times with RPMI, and nuclear retention of the GR in the absence or presence of FK506 was assessed by confocal microscopy. FK506 had no discernable effects on nuclear shuttling of the GR, during either hydrocortisone exposure or wash out (Fig1D and E). Taken together, these observations suggest that the effects of FK506 on innate inflammatory responses to AF in the lung are independent of steroids and map to the innate immune system.

### Calcineurin inhibition leads to impaired cytokine responses, delayed neutrophil recruitment and reduced fungal killing during pulmonary aspergillosis

**Figure 2:**
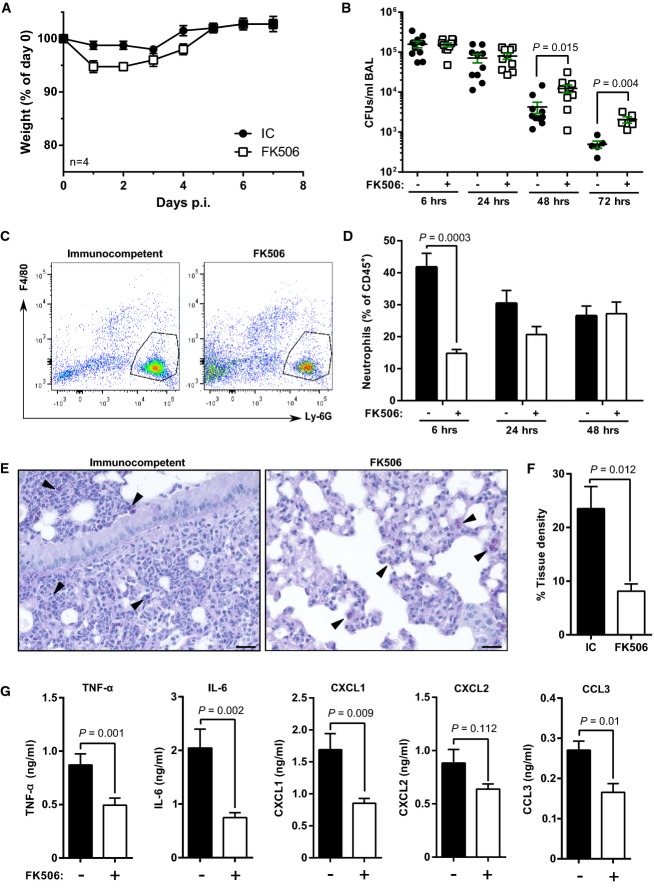
Calcineurin inhibition leads to impaired cytokine production, reduced early neutrophil recruitment and delayed fungal killing during pulmonary aspergillosis Immunocompetent (IC) or FK506-treated (5 mg/kg/day) C57BL/6 WT mice were infected with AF CEA10 (1 × 10^7^ conidia/mouse) i.n.
A Weight was monitored daily for 7 days p.i. Immunosuppressed mice showed increased weight loss through days 1–3 p.i. Data points represent mean ± SEM.B BALs were performed over a 3-day period p.i. and fungal killing assessed by enumerating CFUs. Fungal killing was significantly impaired in immunosuppressed mice. Error bars indicate SEM.C, D Flow cytometry analysis of the CD45^+^ leucocyte populations in BAL. Representative dot plots for CD45^+^ cells (C) and percentages of Ly-6G-positive neutrophils infiltrating the infected lungs over a 48-h p.i. time course (D). The bars indicate the mean percentage (± SEM) of neutrophils of total CD45^+^ leucocytes (*n *=* *5 mice per group). Neutrophil influx was significantly impaired in immunosuppressed mice at 6 h p.i.E PAS-stained lung sections of immunocompetent and FK506-treated animals at 6 h p.i. Scale bar: 50 μm. IC animals showed inflammatory cell infiltrates around conidia (black arrows), whereas in FK506-treated animals, only uptake of conidia by the resident macrophage population could be seen.F Tissue density of whole lung sections was quantified by pixel intensity analysis using ImageJ. Bars represent mean percentages (± SEM), *n *=* *3.G Luminex multiplex cytokine analysis of BAL SNs at 6 h p.i. Bars represent mean ± SEM.
Data information: For statistical analysis, individual time points were compared using Student's *t*-test.

To characterize the effects of calcineurin inhibition on innate immune responses, we established a hydrocortisone-free model of pulmonary aspergillosis based on FK506 monotherapy. Mice were immunosuppressed with FK506 and challenged intranasally with 10^7^ AF resting conidia. Both control and FK506-treated animals were able to survive high-dose challenge with AF. However, FK506-treated animals exhibited increased weight loss and impaired clearance of conidia (average of 4.1-fold higher CFUs) from the airway by 72 h post-infection (Fig2A and B). Time-course FACS analysis revealed that early neutrophil influx was significantly impaired in the airway in response to infection (Fig2C and D). Similarly, histopathological analysis showed cellular recruitment to foci of infection in immunocompetent, but not FK506-treated animals (Fig2E), and consequently reduced tissue density at a whole lung level (Fig2F). Luminex multiplex analysis of airway-based responses indicated that early (6 h p.i.) TNF-α, IL-6, CXCL1 and CCL3 responses to fungal challenge were significantly attenuated in FK506-treated animals (Fig2G). In addition, we observed similar defects in AF clearance, neutrophil recruitment and cytokine responses in RAG^−/−^ mice treated with FK506 (Supplementary Fig S1). Collectively, these observations suggest that FK506 immunosuppression results in impaired early innate cytokine responses, leading to impaired neutrophil recruitment and fungal killing.

### Calcineurin inhibition impairs neutrophil recruitment to the site of infection in a zebrafish model of aspergillosis

**Figure 3:**
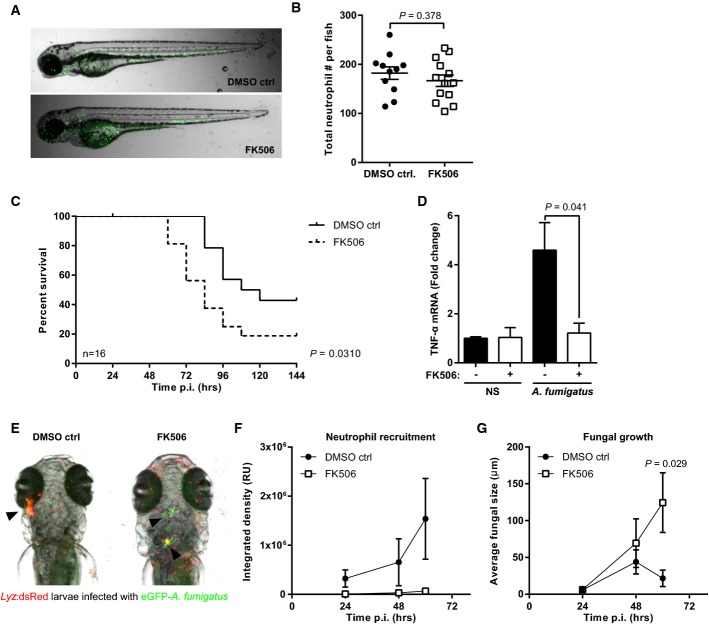
Calcineurin inhibition impairs neutrophil recruitment in a zebrafish model of invasive aspergillosis At 2 days post-fertilization (dpf), *Lyz*:dsRed or *mpx*:GFP larvae were transferred into DMSO ctrl or FK506 (1 μg/ml) containing 0.5 × E2. At 3 dpf, zebrafish larvae were microinjected with AF eGFP conidia into the hindbrain and mortality, and fungal burden and neutrophil recruitment were assessed.
A, B Absolute neutrophil numbers in treated and untreated *mpx*:GFP larvae were quantified by direct counting of fluorescent neutrophils in non-infected whole fish (*n *=* *11-13 per group). There were no significant differences.C For survival analysis, *lyz*:dsRed larvae were microinjected with ˜10 conidia and the effects of FK506 on the outcome from invasive aspergillosis was determined by Kaplan–Meier analysis for survival over 6 days post-infection (*n *=* *16 per group). Immunosuppression led to a significant increase in mortality.D TNF-α mRNA levels were measured in whole non-infected or infected larvae 48 h p.i. Immunosuppression led to significant and almost complete inhibition of TNF-α expression during infection.E-G *Lyz*:dsRed larvae were infected with ˜50 eGFP-expressing conidia of *A. fumigatus* and were imaged daily from 24 h p.i. (E) The effects of FK506 on neutrophil recruitment were assessed by integrated density analysis of red fluorescent neutrophils at sites of infection in ImageJ. A representative image showing impaired neutrophil recruitment to the site of infection in immunosuppressed larvae is shown (60 h p.i.). The arrows indicate infectious foci. (F) Neutrophil recruitment was minimal throughout the first 60 h p.i. in immunosuppressed larvae (*n *=* *8 per group). (G) Fungal growth was determined by measurement of the length of individual fluorescent hyphal elements (*n *=* *8 per group).
Data information: All data represent mean ± SEM and were statistically analysed by Student's *t*-test.

Zebrafish have emerged as an ideal model for the direct visualization of innate immune responses during infection (Henry *et al*, 2013), and calcineurin is highly conserved between man and zebrafish (Supplementary Table S1). To clearly visualize the effects of calcineurin inhibition on leucocyte trafficking during early fungal infection at a cellular level *in vivo*, we established a new high-mortality zebrafish model of aspergillosis. Zebrafish larvae showed AF dose-dependent mortality after hindbrain infection. Microinjection of 10 RC resulted in 20% mortality and 50 RC resulted in 100% mortality by 168 h post-infection (Supplementary Fig S2A). We first characterized macrophage and neutrophil responses to AF infection in zebrafish larvae harbouring red macrophages (*mpeg*:mCherry) or red neutrophils (*lyz*:dsRed). Strong macrophage recruitment was seen from the early stages of infection (Supplementary Fig S2B), whereas neutrophils were only recruited after conidia had started to germinate (Supplementary Fig S2C). We then studied the effects of FK506 on survival and neutrophil recruitment in response to infection. Although FK506 treatment had no effect on resting total neutrophil numbers (Fig3A and B), it lead to significantly increased mortality from invasive aspergillosis (Fig3C). Increased mortality was associated with impaired TNF-α responses, reduced neutrophil recruitment to the site of infection and increased hyphal growth (Fig3D–G). Additionally, FK506 impaired neutrophil recruitment to injury in a tail-cut model (Supplementary Fig S3). Taken together, these findings strongly suggest that calcineurin plays a central role in neutrophil recruitment during acute inflammatory responses in vertebrates.

### FK506 impairs calcineurin–NFAT-dependent TNF-α responses to *A. fumigatus* in murine macrophages

As we saw an increased pulmonary fungal burden in the airway, we assessed the ability of murine macrophages and neutrophils to kill AF *in vitro*. FK506 did not significantly impair the ability of murine macrophages to phagocytose conidia, produce reactive oxygen species in response to infection, or inhibit fungal growth (Supplementary Fig S4A–C). Similarly, no killing defect was observed in FK506-treated neutrophils (Supplementary Fig S4D).

**Figure 4:**
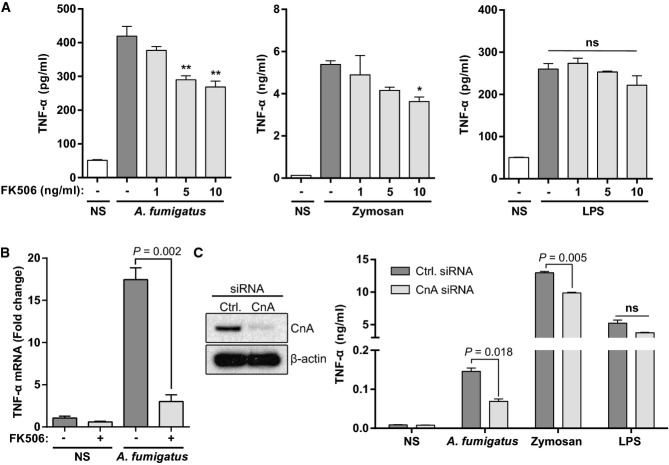
FK506 impairs calcineurin-dependent TNF-α responses to *A. fumigatus* in murine macrophages
A J774A.1 macrophages were treated with an increasing concentration of FK506 and stimulated with AF SC (MOI = 1), zymosan (50 μg/ml) or LPS (25 ng/ml) overnight. TNF-α was measured in the culture supernatant by ELISA. FK506 significantly reduced TNF-α levels at 5 and 10 ng/ml for AF. A significant reduction in TNF-α was also seen for zymosan, but not LPS.B J774A.1 cells were treated with FK506 (10 ng/ml) and either left non-stimulated (NS) or stimulated with AF SC (MOI=1) for 6 h. TNF-α mRNA levels were quantified from whole-cell RNA extracts by RT–PCR. FK506 significantly reduced TNF-α expression.C J774A.1 macrophages were treated with control (Ctrl.) or calcineurin A (CnA)-targeting siRNA (50 nM) and stimulated with either AF SC (MOI = 1), zymosan (50 μg/ml) or LPS (25 ng/ml) overnight. TNF-α was measured in the culture supernatant by ELISA. CnA siRNA led to significant reductions in both AF- and zymosan-dependent TNF-α levels, but had no significant effect on LPS responses.
Data information: Multiple comparisons for (A) were performed after a two-way ANOVA, and comparison for (B, C) was carried out by Student's *t*-test. ns, not significant; NS, non-stimulated. *P < 0.05; **P < 0.01. Data represent mean + SEM of three independent experiments.Source data are available online for this figure.

Alveolar macrophages are critical for mediating sentinel immune responses in the airway. As airway inflammation was impaired *in vivo* and macrophages were the primary immune cell interacting with conidia at early time points after infection (Fig2E and Supplementary Fig S5), we assessed the effects of FK506 on macrophage-based inflammatory responses *in vitro*. Consistent with *in vivo* observations, pre-treatment of murine macrophages with FK506 led to a dose-dependent impairment of TNF-α production in response to challenge with AF SC and zymosan particles (yeast particles that activate TLR2 and Dectin-1) (Fig4A and Supplementary Fig S6A). However, FK506 had no significant effect on LPS-induced TNF-α secretion (Fig4A and Supplementary Fig S6A). In contrast, the NF-κB inhibitor SC514 efficiently inhibited AF, zymosan and LPS-induced TNF-α responses (Supplementary Fig S6B and C). FK506 strongly reduced TNF-α mRNA levels in response to AF infection (Fig4B). This suggested a direct effect of FK506 on calcineurin–NFAT responses. We confirmed the role of calcineurin in TNF-α responses, by siRNA knock-down of the catalytic calcineurin A (CnA) subunit, thereby excluding off-target effects of FK506 (Fig4C).

**Figure 5:**
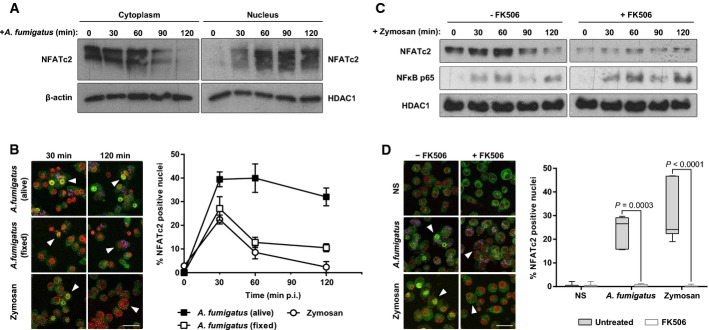
*Aspergillus fumigatus* induces sustained NFAT responses in murine macrophages
A J774A.1 macrophages were stimulated with live AF SC (MOI=5), and macrophage cytoplasmic and nuclear extracts were probed by Western blot for NFATc2. NFATc2 shuttling to the nucleus was observed and was maximal at 2 h p.i.B J774A.1 macrophages were stimulated with live or fixed swollen conidia (MOI = 5) or zymosan (50 μg/ml), and NFAT translocation was assessed by confocal microscopy. NFAT is indicated in green, the nuclei are indicated in red, and AF conidia can be seen in pink. Live conidia were observed to stimulate sustained nuclear translocation compared to dead conidia or zymosan. Data represent mean ± SEM. Scale bar: 50 μm. Arrows indicate *A. fumigatus* or zymosan-positive cells.C J774A.1 macrophages were stimulated with zymosan after FK506 (10 ng/ml) or control treatment, and macrophage nuclear extracts were probed by Western blot for NFATc2 and NF-κB translocation. NFATc2 and NF-κB nuclear translocation was observed, which was maximal at 1 h p.i., in stimulated macrophages. FK506 specifically inhibited NFATc2 translocation, but had no effect on NF-κB.D J774A.1 macrophages were pre-treated with FK506 (10 ng/ml) or vehicle and stimulated with live swollen conidia or zymosan. NFATc2 translocation was evaluated 30 min after stimulation. Box and whisker plot shows median ± maximum/minimum. Scale bar: 50 μm. FK506 completely inhibited nuclear translocation of NFATc2 in response to AF or zymosan. Statistical analysis was carried out by Student's *t*-test. NS, non-stimulated. Arrows indicate *A. fumigatus* or zymosan-positive cells.
Source data are available online for this figure.

To demonstrate direct activation of the calcineurin pathway by AF, we assessed NFATc2 nuclear translocation by Western blotting and confocal microscopy. NFATc2 translocation in response to SC was apparent as early as 30 min after infection and sustained over a 2-h period (Fig5A and B). In contrast, zymosan or fixed SC induced only transient NFATc2 translocation, suggesting that pathogen viability is required to induce persistent NFAT activation (Fig5B). Consistent with calcineurin dependency for the observed NFAT responses, pre-treatment of macrophages with FK506 lead to complete inhibition of AF-dependent and zymosan-dependent NFAT translocation (Fig5C and D). Importantly, NF-κB translocation was not affected (Fig5C). The results from these *in vitro* studies support our *in vivo* studies and indicate that calcineurin–NFAT signalling contributes to the early macrophage inflammatory response during fungal infection. They further demonstrate that collaboration of NF-κB and NFAT signalling pathways is required for maximal inflammatory responses to AF.

### *Aspergillus fumigatus* activates NFAT through a Dectin-1–Syk and Myd88-independent pathway

**Figure 6:**
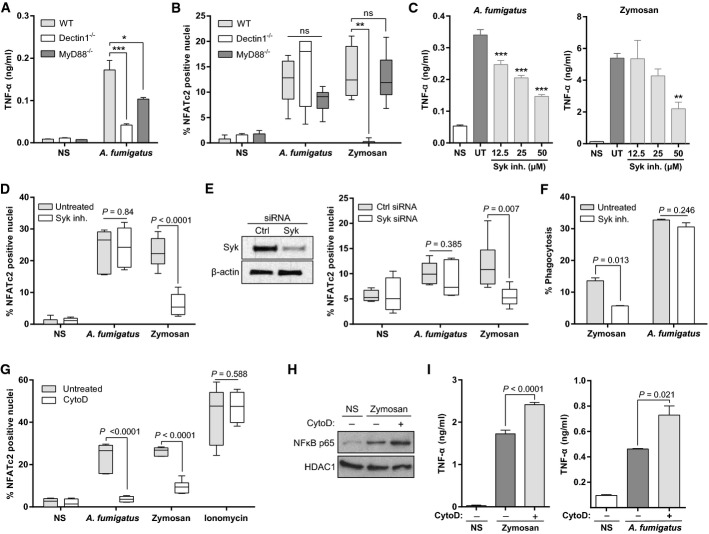
Phagocytosis is required for *A. fumigatus*-induced NFAT activation through a Dectin-1–Syk- and MyD88-independent pathway
A BMDMs from WT, Dectin-1^−/−^ or MyD88^−/−^ mice were infected with swollen conidia (MOI = 1), and TNF-α was measured in the SN after overnight incubation. TNF-α levels were significantly reduced in Dectin1^−/−^ and Myd88^−/−^ BMDMs.B BMDMs were stimulated with swollen conidia (MOI = 5) or zymosan (50 μg/ml), and NFATc2 translocation was quantified by confocal microscopy 30 min after stimulation. Whilst almost complete abrogation of NFAT translocation was seen in Dectin1^−/−^ macrophages stimulated with zymosan, NFAT responses to AF were unaffected.C-E J774A.1 macrophages were pre-treated with (C, D) the Syk inhibitor piceatannol or (E) a control or Syk-targeting siRNA (10 nM) before stimulation with swollen conidia (MOI = 1) or zymosan (50 μg/ml). (C) TNF-α was measured in the SN after overnight incubation. Syk inhibition significantly reduced swollen conidia-dependent or zymosan-dependent TNF-α levels. (D) NFATc2 translocation was quantified by confocal microscopy. Syk inhibition impaired zymosan-dependent NFAT translocation, but did not affect AF-dependent NFAT translocation. (E) Whole-cell lysates of Syk or Ctrl. siRNA-treated cells were separated by SDS–PAGE, followed by Western blotting, and membranes were probed with anti-Syk and anti-β-actin antibodies.F J774A.1 macrophages were pre-treated with piceatannol and infected with swollen conidia (MOI = 5). Phagocytosis was quantified by FACS 30 min after stimulation. Syk inhibition led to impaired phagocytosis of zymosan, but had no effect on phagocytosis of SC.G-I J774A.1 macrophages were pre-treated with cytochalasin D (CytoD, 10 μM) and infected with swollen conidia (MOI = 5) or zymosan (50 μg/ml). (G) NFATc2 translocation after 30 min of stimulation was quantified by confocal microscopy. (H) For quantification of NF-κB translocation, nuclear extracts were separated by SDS–PAGE, followed by Western blotting. Membranes were probed with anti-NF-κB p65 and anti-HDAC1 antibodies. (I) After overnight stimulation, TNF-α was measured in the SN by ELISA. Inhibition of phagocytosis with cytochalasin D led to impaired NFAT translocation in response to zymosan or AF, but not ionomycin (G). In addition, inhibition of phagocytosis lead to increased translocation of NF-κB (H) and increased levels of TNF-α in response to either zymosan or AF.
Data information: Bar graphs represent mean ± SEM and box and whisker plots represent median ± maximum/minimum value. Statistical analysis was performed using (A, B, C) two-way ANOVA for multiple comparisons and (D–I) Student's *t*-test. ns, not significant; *P < 0.05; **P < 0.01; ***P < 0.001. NS, non-stimulated; UT, untreated. Source data are available online for this figure.

As previous studies indicate a role for co-operative TLR and C-type lectin signalling to mediate optimal antifungal responses, next we sought to determine the relative contribution of these pathways to NFAT activation (Gantner *et al*, 2003). We therefore assessed TNF-α responses to zymosan or AF in bone marrow-derived macrophages (BMDMs) from MyD88^−/−^ and Dectin-1^−/−^ mice. Both MyD88^−/−^ and Dectin-1^−/−^ BMDMs exhibited significantly impaired TNF-α production in response to AF SC (Fig6A). NFAT translocation in response to zymosan was completely abrogated in Dectin-1^−/−^ BMDMs, but was not altered in MyD88^−/−^ BMDMs (Fig6B). In contrast, NFAT translocation in response to AF was not impaired in either Myd88^−/−^ or Dectin-1 BMDMs (Fig6B). Taken together, these observations suggest that AF-dependent calcineurin–NFAT activation is both Dectin-1 and Myd88 independent.

To further assess the role of C-type lectin receptors in AF-dependent calcineurin–NFAT activation, we investigated the role of Syk, which acts as the primary tyrosine kinase mediating C-type lectin receptor signalling (Rogers *et al*, 2005; Dennehy *et al*, 2008). Treatment of macrophages with piceatannol, a chemical inhibitor of Syk, impaired both zymosan-induced and AF-induced TNF-α production (Fig6C). However, whilst Syk inhibition resulted in strong inhibition of NFAT translocation in response to zymosan, there was no effect on AF-induced NFAT translocation (Fig6D). To further confirm these observations, we knocked down Syk with siRNA (Fig6E). Consistent with the results obtained with the Syk inhibitor piceatannol, Syk siRNA knock-down leads to a significant reduction in zymosan-induced NFAT translocation, but did not affect AF-induced NFAT translocation. Strikingly, we noted that Syk blockade strongly inhibited zymosan phagocytosis, but had no effect on AF phagocytosis (Fig6F).

### Calcineurin–NFAT activation by *A. fumigatus* is phagocytosis dependent

These results raised the intriguing possibility that macrophage calcineurin–NFAT activation in response to fungi is primarily triggered by phagocytosis. To enable better definition of the relationship between phagocytosis and NFAT translocation, we pre-treated macrophages with cytochalasin D to prevent actin polymerization and phagocytosis. Consistent with our hypothesis, treatment of macrophages with cytochalasin D completely inhibited either zymosan- or AF-induced NFAT activation (Fig6G). Bypass of phagocytosis-dependent NFAT activation with the calcium ionophore ionomycin was not inhibited by cytochalasin D, further demonstrating that the effects of cytochalasin D on NFAT translocation are phagocytosis-related (Fig6G). In contrast, inhibition of phagocytosis with cytochalasin D led to incremental NF-κB translocation in response to zymosan, and higher levels of TNF-α (Fig6H and I).

Taken together, these observations demonstrate that phagocytosis is a prerequisite for fungal NFAT activation in murine macrophages, whereas NF-κB responses do not require uptake. Therefore, these results suggest a bi-modal signalling model in which cell surface-initiated NF-κB signalling is followed by co-operative NF-κB and NFAT responses after internalization.

### TLR9 and Bruton's tyrosine kinase mediate *A. fumigatus*-induced calcineurin activation

**Figure 7:**
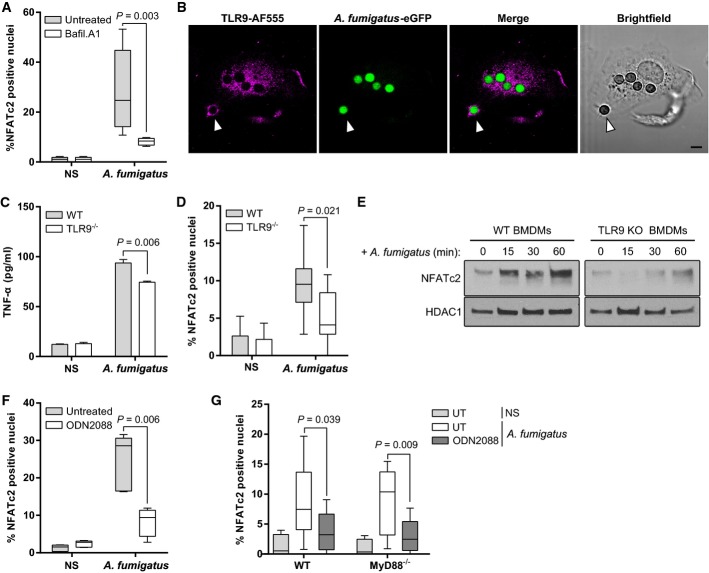
TLR9 mediates MyD88-independent endosomal NFAT activation
A J774A.1 macrophages were pre-treated with bafilomycin A1 (100 nM) and stimulated with swollen conidia (MOI = 3) for 30 min. NFATc2 translocation was assessed by confocal microscopy. Acidification was required for NFATc2 translocation.B TLR9 recruitment was assessed in WT BMDMs 30 min after infection with eGFP-expressing swollen conidia by confocal microscopy. Scale bar: 10 μm. The arrow indicates a TLR9-positive AF-containing phagosome.C BMDMs from WT or TLR9^−/−^ mice were infected with swollen conidia (MOI = 1), and after overnight stimulation, TNF-α was measured in the SN by ELISA. TLR9^−/−^ BMDMs had significantly lower TNF-α levels.D, E BMDMs from WT or TLR9^−/−^ mice were infected with swollen conidia (MOI = 5) for 30 min, and NFATc2 translocation was quantified by (D) confocal microscopy and (E) Western blotting of nuclear extracts. TLR9^−/−^ BMDMs exhibited reduced NFATc2 translocation by both approaches.F J774A.1 macrophages were pre-treated with the TLR9-blocking nucleotide ODN2088 (10 μM) for 30 min, and NFATc2 translocation was quantified by confocal microscopy. This led to a significant reduction in NFATc2 translocation in response to AF.G BMDMs from WT or MyD88^−/−^ mice were pre-treated with ODN2088 (10 μM) and infected with swollen conidia (MOI = 5) for 30 min. NFATc2 translocation was quantified by confocal microscopy. In both, WT and MyD88^−/−^ BMDMs, blocking TLR9 significantly impaired NFATc2 translocation.
Data information: Bar graphs represent mean + SEM and box and whisker plots represent median maximum/minimum value. All statistical analyses were performed using Student's *t*-test. NS, non-stimulated. One representative out of three experiments is shown. Source data are available online for this figure.

To investigate the interplay between phagosomal maturation and calcineurin activation, we first characterized the maturation process of AF-containing phagosomes. Phagosomes containing SC rapidly acquired the early endosomal marker Rab5 and the mid-stage phagosomal marker Rab7. Acidification was maximal 30 min after infection (Supplementary Fig S7). Acidification was required for calcineurin activation as the vacuolar-type H^+^-ATPase inhibitor bafilomycin A1 blocked NFAT translocation in response to AF (Fig7A). Endosomal TLRs require acidification for activation (Lee & Barton, 2014), and it has been shown that TLR9 is recruited to the AF-containing phagosome (Kasperkovitz *et al*, 2010). We therefore hypothesized that TLR9 may mediate calcineurin–NFAT signalling in macrophages in response to AF. We confirmed that TLR9 is recruited to the AF-containing phagosome within 30 min after internalization in BMDMs (Fig7B) and that TLR9 partially contributed to AF-induced TNF-α responses (Fig7C). BMDMs from TLR9^−/−^ mice showed impaired AF-induced NFATc2 translocation (Fig7D and E) and, additionally, the TLR9 blocking nucleotide ODN2088 blocked NFATc2 translocation in J774A.1 macrophages without blocking conidial uptake (Fig7F and Supplementary Fig S8A).

Our studies with AF indicated that NFATc2 translocation was not dependent on MyD88 (Fig6B). We therefore assessed the dependency of TLR9 signalling via calcineurin on the MyD88 pathway. Inhibition of TLR9 signalling in Myd88^−/−^ BMDMs with ODN2088 resulted in an equivalent reduction in NFATc2 translocation to that observed in wild-type BMDMs (Fig7G). Taken together, these observations indicate that phagosome acidification and TLR9 signalling are required for calcineurin activation in response to AF and through a MyD88-independent pathway.

**Figure 8:**
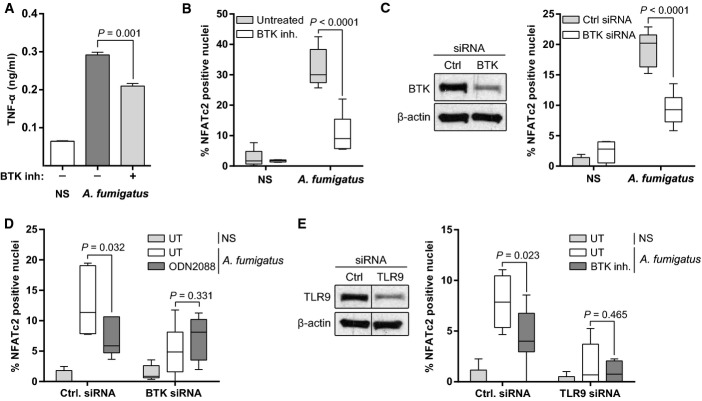
Bruton's tyrosine kinase mediates TLR9-dependent calcineurin activation
A, B J774A.1 macrophages were pre-treated with the BTK inhibitor LFM-A13 (12.5 μM) and infected with swollen conidia. (A) After overnight stimulation, TNF-α was measured in the SN by ELISA. (B) After 30 min of stimulation, NFATc2 translocation was quantified by confocal microscopy. Both TNF-α levels and NFATc2 translocation were significantly reduced by BTK blockade.C J774A.1 macrophages were treated with a control or BTK-targeting siRNA (25 nM), and NFAT translocation was quantified by confocal microscopy. BTK knock-down led to a significant reduction in NFATc2 translocation in response to AF.D, E J774A.1 macrophages were treated with a control, BTK (25 nM)- or TLR9 (75 nM)-targeting siRNA and additionally pre-treated with either ODN2088 (10 μM) or the BTK inhibitor LFM-A13 (12.5 μM). The cells were infected with swollen conidia (MOI = 5), and NFATc2 translocation was measured by confocal microscopy. Blocking both TLR9 and BTK signalling had no additive effect on NFAT translocation blockade.
Data information: Bar graphs represent mean ± SEM and box and whisker plots represent median ± maximum/minimum value. All statistical analyses were performed using Student's *t*-test. NS, non-stimulated. Source data are available online for this figure.

Bruton's tyrosine kinase (BTK) is a key activator of calcineurin in B cells (Antony *et al*, 2004) and TLR9 signalling as been directly linked to BTK activation (Lougaris *et al*, 2014). We therefore hypothesized that BTK signalling contributes to AF-induced calcineurin activation in macrophages. Inhibiting BTK with the selective chemical inhibitor LFM-A13 demonstrated that BTK signalling was required for competent TNF-α responses and calcineurin–NFAT activation in macrophages (Fig8A and B). This finding was further validated by BTK-specific siRNA knock-down (Fig8C). Both the BTK inhibitor and treatment with BTK-targeting siRNA did not affect uptake of AF conidia (Supplementary Fig S8B and C). Blockade of TLR9 signalling with ODN2088 in BTK siRNA knock-down macrophages did not lead to a further reduction in NFATc2 translocation in response to AF (Fig8D). In addition, chemical inhibition of BTK in TLR9 siRNA knock-down macrophages did not result in a further reduction in NFATc2 translocation in response to AF (Fig8E). Taken together, these observations suggest that TLR9-dependent BTK signalling leads to calcineurin–NFAT activation after phagocytosis of AF.

### Calcineurin–NFAT signalling is required for maximal TNF-α responses in alveolar macrophages from lung transplant recipients

**Figure 9:**
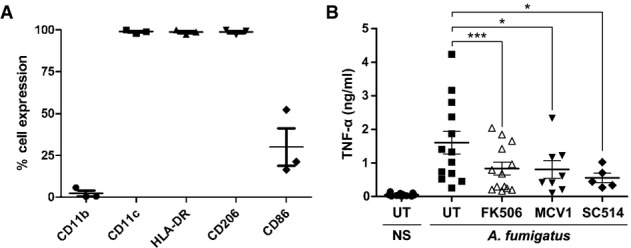
TNF-α responses to *A. fumigatus* in human alveolar macrophages are dependent on both NF-κB and NFAT signalling Alveolar macrophages from 13 different lung transplant recipients were isolated from bronchoalveolar lavage. Cells were pre-treated for 1 h with FK506 (10 ng/ml), the NFAT inhibitor MCV1 (1 μM) or SC514 (10 μM) before stimulation with *A. fumigatus* swollen conidia (MOI 0.1). After 24 h of stimulation, TNF-α was measured in the cell culture supernatant by ELISA.
A Representative FACS-phenotyping demonstrative of a normal alveolar macrophage phenotype is shown for three patient samples.B Inhibition of alveolar macrophage responses to AF with FK506, MCV1 or SC514 led to a significant reduction in TNF-α levels.
Data information: Data represent mean ± SEM. Statistical test used is Kruskal–Wallis with Dunn's multiple comparison. *P < 0.05, ***P < 0.001. Each data point represents an individual donor.

Lung transplant recipients are at high risk of pulmonary aspergillosis (Baddley *et al*, 2010). In order to clinically validate our findings from murine studies, we sought to characterize fungal calcineurin-dependent responses in alveolar macrophages from lung transplant recipients. Alveolar macrophages were isolated from 13 patients undergoing bronchoscopic surveillance, washed and isolated by adherence to plastic. Cells were subcultured for 3 days in standard media, refreshed and calcineurin–NFAT antifungal responses investigated. Cell purity and phenotype were confirmed by FACS analysis (Fig9A).

Consistent with our observations in murine models, FK506 significantly inhibited TNF-α responses in human alveolar macrophages (Fig9B). In addition to FK506, we used the short peptide inhibitor of calcineurin–NFAT signalling MCV1 and the IKK2 inhibitor SC514 to assess the relative contribution of NFAT and NF-κB signalling to TNF-α responses to AF in human alveolar macrophages. In keeping with our murine studies, we observed that both NFAT- and NF-κB-dependent signalling pathways contributed to the inflammatory response to AF in human alveolar macrophages (Fig9B).

## Discussion

The sensing of pathogens by pattern recognition receptors is critical for both co-ordination of effective innate immune responses and control of infection (Romani, 2011). For opportunistic fungal pathogens such as *C. albicans* and AF, collaboration between TLRs and C-type lectins is required for the generation of optimal inflammatory responses via NF-κB (Romani, 2011). We and others have recently shown that generation of calcineurin–NFAT-dependent immune responses is important for early sterilization of both fungal and bacterial infections (Greenblatt *et al*, 2010; Fric *et al*, 2012; Herbst *et al*, 2013; Tourneur *et al*, 2013). However, until now, the mechanistic basis for integration of NFAT- and NF-κB-dependent immune responses was poorly understood. In the present study, we demonstrate that phagocytosis-dependent calcineurin–NFAT signalling in macrophages co-operates with cell surface NF-κB-mediated signalling to potentiate TNF-α-driven innate immune responses to AF. NF-κB signalling occurs independently of phagocytosis, but phagocytosis is critical for activation of NFAT-dependent inflammatory pathways. These observations highlight a crucial new mechanism by which the host is able to discriminate intracellular from extracellular signals and co-ordinate appropriate and co-operative immune responses.

Our current understanding is that the NFAT family of transcription factors arose around the time that vertebrates emerged, around 500 million years ago (Wu *et al*, 2007). The NFAT family is most likely to be derived from a recombination event that fused a calcium-responsive element analogous to the zinc finger transcription factor Crz1 in fungi, and a Rel homology domain present in NF-κB (Wu *et al*, 2007). This recombination event enabled integration of calcium signals for vertebrate-specific organogenesis and adaptive immune responses. The calcineurin–NFAT system has been shown to be a key sensing system across a number of cell types that is able to integrate a range of external signals that result in an increase in intracellular calcium levels (Muller & Rao, 2010). This leads to the activation of calcineurin phosphatase, subsequent dephosphorylation of NFAT and NFAT nuclear import, which is a dynamic process, with shuttling out of the nucleus mediated by a number of kinases (Im & Rao, 2004). Recent studies have demonstrated that different isoforms of NFAT exhibit dynamic response diversity, depending on the kinetics of calcium flux (Yissachar *et al*, 2013). Within the context of pathogen sensing, this raises the possibility that the calcineurin–NFAT pathway may enable physical discrimination of organisms based on the kinetics of host–pathogen interactions, leading to a transcriptional program that co-ordinates systems-level vertebrate immune responses.

Our studies with the model mould pathogen AF clearly indicate that macrophage NFAT activation is dependent upon phagocytosis and that sustained activation occurs with live organisms. Furthermore, we noted that conidial germination was a prerequisite for NFAT activation, indicative of a requirement for fungal ligand exposure as well as pathogen internalization for pathway activation. We found that inhibition of phagocytosis leads to complete inhibition of NFAT translocation but increased NF-κB translocation, resulting in a net increase in TNF-α production. This finding is consistent with earlier studies of “frustrated phagocytosis”, demonstrated for *C. albicans*, and further extend our understanding of how NFAT and NF-κB integrate to mediate innate immune responses (Rosas *et al*, 2008). Surface ligation of pattern recognition receptors seems to primarily lead to NF-κB activation, whereas for signalling from the endosome, there is collaboration between NFAT and NF-κB. Notably, NFAT is known to negatively regulate both itself and NF-κB via the protein RCAN1 (regulator of calcineurin 1) (Wu *et al*, 2013) and has been shown to have specific binding sites in the TNF-α promoter (Oum *et al*, 2002). Future studies will aim to comprehensively identify all of the genes that are under the control of NFAT and NF-κB during AF infection of macrophages.

**Figure 10:**
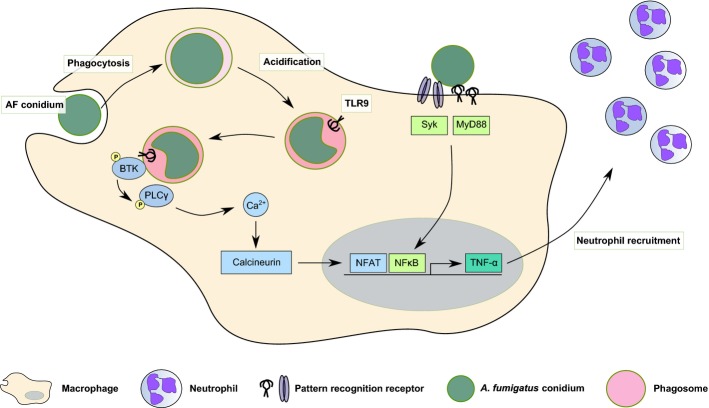
Proposed model for the integration of NFAT and NF-κB signalling pathways Swollen AF conidia are taken up by macrophages. The AF-containing phagosome rapidly acidifies, resulting in the activation of endosomal PRRs such as TLR9. TLR9 signalling drives BTK activation, which in turn activates phospholipase Cγ (PLCγ) by phosphorylation. PLCγ activation initiates the calcium flux required for calcineurin-mediated NFAT translocation into the nucleus. Additionally, NF-κB translocation is activated from the cell surface and acts in synergy with NFAT for TNF-α production. TNF-α secretion results in neutrophil recruitment which is the cell type required for efficient fungal clearance.

Our *in vivo* studies suggest that phagocytic activation of NFAT in macrophages is critically important for early cytokine and chemokine release in the airway and rapid recruitment of neutrophils (summarized in Fig10). Further direct visualization of leucocyte trafficking in zebrafish models of invasive aspergillosis and tail-cut trauma enabled independent confirmation of the principal findings from our murine studies. This approach revealed a general role for calcineurin signalling in neutrophil recruitment to sites of infection or sterile inflammation. Our zebrafish model further confirmed that the antifungal effect of FK506 which we have previously demonstrated (Herbst *et al*, 2013) is clearly outweighed by a failure to recruit neutrophils to sites of infection *in vivo*. This leads to a net increase in susceptibility to invasive aspergillosis. Our observations therefore underscore the primary importance of neutrophils for immunity to AF (Baddley *et al*, 2010).

These findings are in keeping with previous studies of invasive candidiasis in which calcineurin signalling was shown to be required for neutrophil-mediated killing (Greenblatt *et al*, 2010), but they also suggest that for pulmonary aspergillosis, impaired macrophage-dependent recruitment of neutrophils is the primary mechanism that leads to impaired immunity. In previous studies, we observed a calcineurin-dependent killing defect in MH-S murine macrophages in response to AF. However, we have subsequently found that these cells do not activate calcineurin–NFAT signalling in response to AF. In contrast, both bone marrow-derived macrophages and J774A.1 murine macrophages activate calcineurin–NFAT signalling in response to AF and exhibit calcineurin-dependent cytokine and chemokine responses, but with no defect in fungal killing. Taken together, these observations suggest that calcineurin signalling contributes to a number of distinct innate antifungal responses that are both fungal species and myeloid cell specific.

For zymosan and *C. albicans,* calcineurin–NFAT activation is mediated through Dectin-1 (Goodridge *et al*, 2007). However, the molecular basis for Dectin-1-dependent calcineurin activation is currently poorly understood. We show that calcineurin–NFAT activation and phagocytosis of zymosan are dependent on Dectin-1 and Syk. However, we found that whilst Dectin-1 and Syk appeared to be important for AF-dependent TNF-α production, Dectin-1 or Syk was not required for AF-dependent phagocytosis or NFAT translocation. These findings highlight the differences between the recognition of *C. albicans* and AF, and raise the possibility that calcineurin activation in response to zymosan or *C. albicans* is a secondary effect of Dectin-1-dependent phagocytosis. Interestingly, further studies with particulate LPS indicate that CD14 endocytosis also leads to calcineurin–NFAT signalling (Zanoni *et al*, 2009). The emerging picture is of a two-step process whereby irrespective of the mode of internalization, phagosomal maturation leads to calcineurin–NFAT activation after the internalization of AF.

For AF, we observed that phagocytosis and phagosomal acidification were required for the activation of TLR9–BTK–calcineurin–NFAT signalling (Fig9). We further noted that whilst TLR9–BTK–calcineurin–NFAT signalling was phagocytosis dependent, none of these components appeared to be required for AF phagocytosis. These observations are consistent with the finding that TLR9 localizes to the AF phagosome and is required for immunity and inflammatory responses to AF in animal models (Ramaprakash *et al*, 2009; Kasperkovitz *et al*, 2010). Recent studies indicate that both fungal cell wall chitin and fungal DNA contribute to *Candida* TLR9 activation (Ramirez-Ortiz *et al*, 2008; Wagener *et al*, 2014). Furthermore, BTK is also recruited to the *Candida* phagosome (Kubo *et al*, 2009; Strijbis *et al*, 2013). This raises the possibility that BTK may also contribute to *Candida*-dependent activation of NFAT, downstream of Syk. Strikingly, we noted that TLR9–BTK–calcineurin signalling was independent of MyD88. However, it has been shown that TLR9 collaborates with MHC class II and BTK to sustain signalling through both MyD88- and TRIF-regulated pathways (Liu *et al*, 2011). Further studies are required to fully define the role TLR9 and BTK in calcineurin–NFAT activation during phagocytosis of fungal and bacterial pathogens and to determine the contribution of other endosomal receptors and downstream signalling molecules.

Our current understanding of the phagocytic uptake of AF is that for dendritic cells, DC-SIGN may be involved in the internalization of conidia, but the role of Dectin-1 is less clear (Serrano-Gomez *et al*, 2005). Recent studies in murine alveolar macrophage cell lines showed that the effects of either Dectin-1 or mannose receptor blockade were modest in terms of AF uptake, consistent with our findings (Slesiona *et al*, 2012). Secreted opsonins such as pentraxin 3, surfactant protein D and complement factor 3 further contribute to conidial uptake (Madan *et al*, 1997; Moalli *et al*, 2010; Schelenz *et al*, 2013). Taken together, these observations suggest that phagocytosis of AF is dependent on a number of complementary mechanisms that may vary depending on cellular phenotype and environment. Further studies are required to determine whether the mode of AF uptake influences subsequent activation of NFAT via TLR9–BTK.

Of the cytokines and chemokines identified by our *in vivo* studies, TNF-α has been shown to be critical for anti-fungal immunity and is a key cytokine required for neutrophil recruitment in murine models of pulmonary aspergillosis (Mehrad *et al*, 1999; Schelenz *et al*, 1999). Our clinical studies further identify a key defect in TNF-α in organ transplant recipients at risk of fungal disease (Armstrong-James *et al*, 2012). Previous studies have shown that both NF-κB and NFAT bind to the TNF-α promoter (Macian *et al*, 2000), suggesting co-operation between these transcription factors. We therefore reasoned that further study of the dependency of antifungal TNF-α responses on NF-κB and NFAT was warranted. This approach enabled us to dissect the principle mechanisms by which NF-κB- and NFAT-dependent antifungal response pathways integrate, using TNF-α as the most robust and clinically relevant marker of innate fungal immunity. We found that impaired macrophage calcineurin-dependent cytokine responses contribute to the failure to recruit neutrophils into the infected lungs. A role for macrophage-dependent chemotaxis is supported by the finding that neutralizing TNF-α during AF infection reduces neutrophil recruitment and delays fungal clearance (Schelenz *et al*, 1999). These observations have wider implications for organ transplant recipients, who are susceptible to a range of infectious and non-infectious chronic inflammatory conditions. A better understanding of the other effector functions that are under the control of NFAT and which are specifically activated by pathogen internalization is now required.

In summary, we have shown that the calcineurin inhibitor FK506 impairs immunity to pulmonary aspergillosis by specifically inhibiting NFAT-dependent innate immune responses in macrophages. This leads to impaired generation of early TNF-α and chemokines in the lung and a delay in neutrophil recruitment that leads to an increased fungal burden. Furthermore, we show that phagocytosis is an essential prerequisite for NFAT activation in response to AF and zymosan, and we establish a novel link between MyD88-independent phagosomal TLR9–BTK signalling and calcineurin activation. These observations identify a new Toll-dependent mechanism by which the host can distinguish intracellular from extracellular-derived signals, in order to generate qualitatively different innate immune responses.

## Materials and Methods

### Fungal strains and culture

AF CEA10 was used for murine infection experiments, and an eGFP-expressing strain (ATCC46645-eGFP, a kind gift from Frank Ebel) was used for all other experiments (Meier *et al*, 2003). All strains were cultured on Sabouraud dextrose agar (Oxoid). Conidia were harvested in 0.1% Tween/H_2_O and filtered through MIRACLOTH (Calbiochem, UK). Conidial suspensions were washed twice in PBS (Baxter Healthcare, UK) and resuspended in phosphate-buffered saline (PBS) at the indicated concentrations. For zebrafish infection studies, conidia were resuspended in PBS containing 1% polyvinylpyrrolidone (Sigma, UK). To generate swollen conidia (SC), resting conidia (RC) were suspended in RPMI at 3 × 10^6^ conidia/ml and swollen at 37°C for 4 h. Fixed SC were generated by fixing in 2% formalin for 30 min at 4°C.

Biotinylation was achieved by resuspending SC in 50 mM NaHCO_3_, pH 8.3, followed by the addition of sulfo-NHS-LC-biotin (Life Technologies). Biotinylation was allowed to proceed for 2 h at 4°C whilst constantly shaking, followed by quenching in 100 mM Tris–HCl, pH 8 for 40 min at 4°C. The biotinylated conidia were washed twice with PBS and stored at 4°C.

### Murine infection experiments

All mouse experiments were approved by the United Kingdom Home Office and performed in accordance with project license PPL 70/7324. C57BL/6 WT male mice (18–22 g; Harlan Ortech, UK), Rag^−/−^ mice (Mombaerts *et al*, 1992), Dectin-1^−/−^ mice (Saijo *et al*, 2007), MyD88^−/−^ (Eltom *et al*, 2014) and TLR9^−/−^ mice (Hemmi *et al*, 2000) on a C57BL/6 background were housed in individually vented cages with free access to autoclaved food and water. All mice were matched for age and gender. For survival experiments, mice received 125 mg/kg hydrocortisone (Hydrocortistab, Sovereign Medical) by s.c. injection every 3 days and 5 mg/kg FK506 (Prograf, Astellas) by i.p. injection daily. All mice received 1 mg/l tetracycline hydrochloride (Sigma) and 64 mg/l ciprofloxacin (Sigma) in drinking water as prophylaxis against bacterial infections. For all other mouse experiments, mice did not receive hydrocortisone. For infection, mice were anaesthetized by isoflourane inhalation and intranasally inoculated with RC in 40 μl of PBS. For survival studies, mice were culled at 20% weight loss as a UK Home Office established endpoint. For histopathological studies, lungs were fixed in 10% buffered formalin for 24 h and embedded in paraffin. Sections (4 μm) were stained with PAS, and lung inflammation was analysed using ImageJ software (rsb.info.nih.gov/ij/). Lung density was calculated by dividing thresholded lung area by the total lung area. Bronchoalveolar lavage (BAL) was performed by instilling 1 ml of PBS per lung into the trachea after intubation with a 20-g Venflon intravenous catheter (Becton Dickinson, UK).

### Colony-forming unit counts

CFUs in the BAL were estimated by performing a serial dilution of BAL fluid in 0.1% Tween-20/dH_2_O and plating onto Sabouraud agar (Oxoid, UK) plates. Plates were incubated at 37°C overnight, and CFUs were counted the next day.

### Analysis of cell influx into alveolar space

BAL cells were incubated with a Fc receptor blocking antibody (anti-CD16/CD32, clone 93, eBioscience) on ice for 20 min, followed by staining with anti-F4/80-APC/Cy7 (clone BM8, Biolegend), anti-Ly-6G-BV421 (clone 1A8, Biolegend) and anti-CD45-PE/Cy7 (clone 30-F11, eBioscience). Samples were stained for 20 min in the dark, followed by red blood cell lysis in BD FACS lysing solution. After 1 wash in PBS, cells were fixed in 2% paraformaldehyde (PFA) and acquired on a Fortessa flow cytometer.

### Image stream cell analysis

C57BL/6 mice were infected with 1 × 10^7^ resting conidia stained with Calcofluor White (Sigma). Four hours after infection, BALs were performed as described earlier. Cells were stained with CD45-PE (eBioscience) and DRAQ5 (Cell Signaling), and data were acquired on an ImageStreamX (Amnis). Using IDEAS software, cells with in-focus nuclei were chosen for further analysis. Neutrophils and macrophages were identified according to differential intensity of CD45 staining and shape of the nucleus.

### Murine macrophage culture and stimulation

Bone marrow from C57BL/6 WT, MyD88, Dectin-1 and TLR9 knockout mice aged 8–10 weeks was harvested and differentiated into macrophages in RPMI containing 10% heat-inactivated FCS, 50 μM β-mercaptoethanol and 40 ng/ml murine M-CSF (eBioscience) for 7 days. The murine macrophage cell line J774A.1 was cultured in RPMI containing 10% heat-inactivated FCS and 50 μM β-mercaptoethanol at 37°C, 5% humified CO_2_. siRNAs (Ambion) were transfected using the INTERFERin (Polyplus) transfection reagent according to the manufacturer's instructions. The day before stimulation, macrophages were activated with 200 U/ml recombinant murine IFN-γ (R&D Systems) overnight. Macrophages were treated with FK506 (10 ng/ml, Calbiochem), cytochalasin D (1 or 10 μM, Sigma), the Syk inhibitor piceatannol (50 μM, Sigma), the BTK inhibitor LFM-A13 (25 μM, Sigma), the IKK2 inhibitor SC514 (24 μM, Calbiochem), bafilomycin A1 (100 nM, Sigma) or the TLR9 blocking nucleotide ODN2088 (1 μM, InvivoGen) for 1 h prior to stimulation with swollen conidia at the indicated MOIs, zymosan (50 μg/ml, InvivoGen) or LPS (25 ng/ml, InvivoGen).

### Confocal microscopy

Cells seeded on coverslips were fixed in 2% PFA for 15 min, followed by quenching in 50 mM NH_4_Cl for 10 min. The cell membrane was permeabilized in PBS containing 0.2% Triton X-100 and 10% FCS for 15 min, followed by incubation with the primary antibody (anti-NFATc2, clone D43B1, anti-glucocorticoid receptor, clone D8H2; anti-Rab5, clone C8B1; anti-Rab7, clone D95F2; all from Cell Signaling and anti-TLR9, ThermoFisher Scientific) for 1 hr at RT. After washing in PBS, cells were incubated with an anti-rabbit AF555 (Life Technologies) antibody for 45 min at RT and mounted with Vectashield mounting medium containing DAPI (Vector Laboratories). Imaging of acidification using LysoTracker® Red DND-99 (Life Technologies) was performed on live cells. Images were taken on a LSM-510 Zeiss microscope. NFAT and GR translocation was quantified by calculating the Manders coefficient using the JaCOP plugin (Bolte & Cordelieres, 2006) for ImageJ, and colocalization of phagosomal markers was assessed by quantifying the average pixel intensity around single conidia using ImageJ.

### Western blotting

For whole-cell lysates, cells were lysed in 150 mM NaCl, 50 mM Tris (pH 8), 1% Triton X-100 containing proteinase inhibitors (Mini complete, Roche). Cytoplasmic and nuclear extracts were prepared using the NE-PER extraction reagents from Thermo. Extracts were separated by SDS–PAGE, and the proteins were transferred onto nitrocellulose membranes. Membranes were probed with anti-NFATc2 (D43B1), anti-NF-κB p65 (C22B4), anti-HDAC1 (10E2), anti-Pan-calcineurin A, anti-Syk (D3Z1E), anti-BTK (D3H5) and anti-β-actin (8H10D10) antibodies. All antibodies were from Cell Signaling.

### Real-time PCR

A total of 5 × 10^5^ macrophages or five zebrafish larvae were lysed in Tri Reagent (Sigma) and whole RNA extracted according to the manufacturer's instructions. RNA was reverse-transcripted using the QantiTect kit according to the manufacturer's instruction (Qiagen). mRNA levels were quantified using SYBR Green JumpStart system from Sigma. HPRT was used as a reference for murine and EF1α for zebrafish RT–PCRs, and fold change for TNF-α mRNA levels was calculated using the Δ*C*_t_ method.

Primers were as follows:

murine HPRT forward:5′-GTAATGATCAGTCAACGGGGGAC-3′;
murine HPRT reverse: 5′-GCAAGCTTGCAACCTTAACCA-3′;
murine TNF-α forward: 5′-GAGCTTTCAACAACTACTCAG-3′;
murine TNF-α reverse: 5′-GGAAGGCCTGAGATCTTATC-3′;
zebrafish EF1α forward: 5′- AAGCTTGAAGACAACCCCAAGAGC-3′;
zebrafish EF1α reverse: 5′- ACTCCTTTAATCACTCCCACCGCA-3′;
zebrafish TNF-α forward: 5′-AGACCTTAGACTGGAGAGATGAC-3′;
zebrafish TNF-α reverse: 5′-CAAAGACACCTGGCTGTAGAC-3′ (Stoc-khammer *et al*, 2009).

### ELISA and luminex assays

TNF-α and CXCL1 levels were measured in tissue culture or BAL supernatants using DuoSet mouse ELISA kits from R&D Systems. Luminex analysis of BAL supernatants was performed using the multiplex kit from R&D Systems. BAL supernatants were screened for levels of CXCL1, CXCL2, CCL3, IL-6 and TNF-α.

### Phagocytosis assays

Cells were pre-treated with the inhibitors of interest for 1 h, before the addition of 50 μg/ml Alexa Fluor 488-conjugated Zymosan A particles (Life Technologies) or biotinylated SC ATCC46645-eGFP at a MOI of 5. Non-phagocytosed conidia were counterstained with Cy3-labelled streptavidin (VWR). Macrophages were labelled with anti-CD11b (Biolegend), and samples were acquired on a Fortessa flow cytometer.

### Zebrafish care and maintenance

All zebrafish experiments were approved by the United Kingdom Home Office and performed in accordance with the project license PPL 70/7446. Wild-type adult breeders were purchased from the Zebrafish International Resource Center (Eugene, OR), and the Tg(*lyz*:dsRed)^*nz50*^ (referred to as *Lyz*:dsRed), Tg(UAS-E1b:Eco.NfsB.mCherry)^*c24*^ (referred to as *mpeg*:mCherry) and the Tg(mpx:GFP)^ill^ (referred to as *mpex*:GFP) zebrafish lines have been described elsewhere (Renshaw *et al*, 2006; Hall *et al*, 2007; Mostowy *et al*, 2013; Palha *et al*, 2013). Embryos were raised in Petri dishes containing 0.5 × E2 medium supplemented with 0.3 μg/ml of methylene blue. For imaging experiments, from 24 h post-fertilization (hpf), 0.003% 1-phenyl-2-thiourea (Sigma-Aldrich) was added to the medium to prevent melanin synthesis. Embryos were reared at 30°C, and larvae were anaesthetized with 200 μg/ml tricaine (Sigma-Aldrich) during the injection and imaging procedures

### Zebrafish infection experiments and imaging

At 2 days post-fertilization (dpf), zebrafish larvae were transferred into 0.5 × E2 containing 1 μg/ml FK506. The next day, AF ATCC46645-eGFP RC were prepared at a stock concentration of 5 × 10^7^/ml and up to 10 μl was injected into the hindbrain resulting in an inoculum of 10 to 50 conidia per embryo. Infected larvae were kept individually in 24-well plates at 30°C and FK506 remained in the medium throughout the infection period. Survival was assessed every 12 h. Neutrophil and macrophage recruitment and hyphal growth were assessed by taking images with a Leica M205 FA stereomicroscope every 12–24 h, and images were analysed using ImageJ.

### Isolation of human alveolar macrophages

All human studies were approved by the Biomedical Research Unit (Advanced lung disease) Biobank research project (NRES reference 10/H0504/9), Royal Brompton and Harefield NHS Trust (AS1). All experiments conformed to the principles set out in the WMA declaration of Helsinki and the Department of Health and Human Services Belmont Report. Ethical consent was obtained from lung transplant recipients from the Royal Brompton and Harefield NHS Trust due to undergo flexible bronchoscopy. A total of 240 ml saline bronchoalveolar lavage (BAL) was performed by flexible bronchoscopy and transferred on ice for processing. BAL fluid was passed through a 100 μm cell strainer (BD Bioscience, UK) and centrifuged at 400 × *g* for 10 min to pellet cells. The cell pellet was subsequently washed twice in cold PBS and resuspended in warmed RPMI 1640 (as above). Macrophages were plated at the desired concentration and, following incubation for 1 h, a pure adherent alveolar macrophage population was obtained by washing away non-adherent cells twice with warmed RPMI. Alveolar macrophages were then further rested for 3 days to ensure any residual calcineurin inhibitor effect had been removed.

### FACS phenotyping of human alveolar macrophages

Alveolar macrophages were isolated directly from BAL samples and stained with the live-dead stain Zombie-Aqua (Biolegend) according to the manufacturer's instructions, followed by blocking on ice for 20 min with human FcR block (1 in 100 dilution in PBS and 0.1% bovine serum albumin, BD Biosciences). Cells were subsequently stained with antibodies against CD11b (ICRF44), CD11c (3.9), CD206 (15-2), CD86 (IT2.2) and HLA-DR (L234, all Biologend UK Ltd), and samples were acquired on a LSRFortessa (BD Biosciences). Data were analysed with FlowJo Software.

### Human alveolar macrophage stimulation and ELISA

Human alveolar macrophages were plated at 1 × 10^5^ per well in a 96-well plate and pre-treated for 1 h with FK506 (10 ng/ml, Calbiochem), the cell-permeable synthetic maleimido-conjugated NFAT inhibitor MCVI (1 μM, Calbiochem, EMD Millipore) or the NF-κB inhibitor SC-514 (10 μM, Cayman Chemical). Macrophages were incubated with AF SC at an MOI 0.1 for 24 h, and TNF-α release was quantified in culture supernatants using a DuoSet ELISA Development kit from R&D Systems in accordance with the manufacturer's instructions.

### Statistical analysis

All statistical analysis was performed using GraphPad Prism software, two groups were compared by Student's two-tailed *t*-test, and multiple comparisons were performed using one-way analysis of variance (ANOVA). Levels of significance were calculated by Dunnett's multiple comparisons test. Statmate (GraphPad software) was used to estimate the mean differences detectable given a certain number (*n*). For all experiments, at least 80% power was necessary. Expected standard deviations were obtained from set-up experiments. For murine experiments, the sample size estimate for survival experiments was 10. The sample size estimate for neutrophil recruitment by FACS analysis was 6, and the sample size estimate for colony-forming units from bronchoalveolar lavage was 10. For murine survival experiments, animals that showed signs of vestibular infection were excluded from the analysis. Animals were randomly distributed in groups of five per cage, and each cage was assigned a treatment option to avoid interference from coprophagia.

For further details regarding materials and methods, please refer to the Supplementary Information.

## Supporting Information

Supplementary Information

Review Process File

Source Data for Figure 4C

Source Data for Figure 6

Source Data for Figure 7E
