# Supplementary material for: Phagocytosis-dependent activation of a TLR9–BTK–calcineurin–NFAT pathway co-ordinates innate immunity to Aspergillus fumigatus
Source: EMBO Mol Med. 2015 Jan 30;7(3):240–58. doi: 10.15252/emmm.201404556 (PMC4364943; doi:10.15252/emmm.201404556)
Supplement: Supplementary file 1 [file emmm0007-0240-sd1.pdf]

Supplementary information

Contents

Supplementary Figure Legends 2

Supplementary Figure 1: FK506 impairs innate immune responses to *A. fumigatus*. 2

Supplementary Table 1: Calcineurin is highly conserved between humans and zebrafish............ 2

Supplementary Figure 2: Establishment of a zebrafish model of invasive aspergillosis. 2

Supplementary Figure 3: Calcineurin inhibition leads to defective neutrophil recruitment in a zebrafish trauma model of inflammation. 3

Supplementary Figure 4: FK506 does not affect cell intrinsic effector functions. 3

Supplementary Figure 5: Macrophages are the main innate immune cell phagocytosing conidia early after infection. 3

Supplementary Figure 6: NFAT and NFκB contribute to *A. fumigatus*-induced cytokine responses. 3

Supplementary Figure 7: The AF-containing phagosome matures rapidly. 4

Supplementary Figure 8: TLR9 and BTK are not required for *A. fumigatus* phagocytosis in macrophages. 4

Supplementary Figures and Tables 5

Supplementary Figure 1 5

Supplementary Table 1 6

Supplementary Figure 2 7

Supplementary Figure 3 8

Supplementary Figure 4 9

Supplementary Figure 5 10

Supplementary Figure 6 11

Supplementary Figure 7 12

Supplementary Figure 8 13

Supplementary Materials and Methods 14

Antibodies used in this study 14

Supplementary Figure Legends

Supplementary Figure 1: FK506 impairs innate immune responses to *A. fumigatus.*

Rag2-/- mice were immunosuppressed with FK506 (5 mg/kg/day, sc.) and infected i.n. with $1x10^7$ resting conidia *A. fumigatus* CEA10.

(A) FK506 treated animals lost significantly more weight than immunocompetent (IC) controls. n=10, Single time points were compared by Student's *t*-test corrected for multiple comparison using the Holm-Sidak method.

(B-E) BALs were performed 6 hrs and 72 hrs after infection and (B) CFU estimated. FK506 treatment delayed fungal clearance. n=10, p=0.0003. (C) The TNF-α response in BAL supernatant was measured by ELISA. n=5, p<0.0001. (D) Neutrophil recruitment was assessed by FACS analysis. Neutrophils were defined as CD45 positive, Ly-6G positive and F4/80 negative. n=4, p=0.0079. Each dot represents a single animal and lines show mean +/- SEM. IC and FK506 treated groups were compared using Student's *t*-test.

Supplementary Table 1: Calcineurin is highly conserved between humans and zebrafish.

Protein sequence identity of human zebrafish homologues of the isozymes of the calcineurin A and calcineurin B subunit as well as the FKBP12 was estimated using ClustalW2.

Supplementary Figure 2: Establishment of a zebrafish model of invasive aspergillosis.

(A) Survival of *lyz*:dsRed larvae infected with resting eGFP-expressing conidia of *A. fumigatus* was monitored over 7 days p.i. Survival was dose dependent with inocula of ~10 RC per fish resulting in 20% mortality and inocula of ~50 RC per fish resulting in 100% mortality.

(B) *Mpeg*:mCherry larvae were infected with ~50 eGFP-expressing RC and macrophage recruitment was monitored by microscopy. Conidia swelled yet did not germinate in the first 48 hrs p.i. and infection elicited strong macrophage recruitment at both time points, with macrophages and conidia co-localising.

(C) *Lyz*:dsRed larvae were infected with ~50 eGFP-expressing RC and neutrophil recruitment was monitored by microscopy. In the first 48 hrs after infection, no neutrophil recruitment could be detected. Only at 72 hrs p.i., co-incident with fungal germination was strong neutrophil influx seen. At 96 hrs p.i., neutrophils were clearly seen as being adjacent to hyphal structures, however direct co-localisation was rare. Arrows indicate infectious foci and presence or absence of cell recruitment.

Supplementary Figure 3: Calcineurin inhibition leads to defective neutrophil recruitment in a zebrafish trauma model of inflammation.

At 2dpf *mpx*:GFP larvae were transferred into DMSO or FK506 (1 µg/ml) containing 0.5xE2 and incubated over night. At 3 dpf, tail fins were transected. Neutrophil recruitment to the injury was assessed at the indicated time points by stereo microscopy. Neutrophil recruitment was significantly impaired in immunosuppressed larvae. Individual time points were compared by Student's *t*-test corrected for multiple comparison using the Holm-Sidak method, n=8.

Supplementary Figure 4: FK506 does not affect cell intrinsic effector functions.

(A) J774A.1 macrophages were pre-treated with FK506 (10 ng/ml) and infected with swollen conidia at a MOI of 0.1 and fungal growth was assessed by measuring the fungal 18S rRNA adjusted to murine beta-actin by real-time PCR. There were no significant differences.

(B-C) J774A.1 macrophages were pre-treated with FK506 (10 ng/ml) and infected with swollen conidia at a MOI of 5. (B) Phagocytosis was assessed by FACS. (C) ROS production was measured in a plate reader-based assay using 123-Dihydrorhodamine. There were no significant differences.

(D) Murine bone marrow-derived neutrophils were co-incubated with swollen conidia (MOI=0.1) and conidial killing was assessed by CFU after 3 hours of co-incubation. Pre-treatment with FK506 did not affect neutrophil-dependent killing.

Supplementary Figure 5: Macrophages are the main innate immune cell phagocytosing conidia early after infection.

WT C57BL/6 mice were infected with $1x10^7$ Calcofluor White-labelled conidia *A. fumigatus* CEA10 and BALs were performed 4 hours after infection. (A) Macrophages and neutrophils were identified according to their differential intensity of CD45 stain and nuclear shape using Image Stream. (B) The percentage of neutrophils and macrophages in the $CD45^+$/$conidia^+$ gate was determined in 4 mice.

Supplementary Figure 6: NFAT and NFκB contribute to *A. fumigatus*-induced cytokine responses.

(A) WT BMDMs were pre-treated with FK506 (10 ng/ml) and stimulated with AF SC conidia (MOI=3), Zymosan (50 µg/ml) or LPS (25 ng/ml) for 6 hours. Cytokines were measured by ELISA. FK506 significantly impaired TNF-α responses to AF and zymosan but

not LPS. Bars show mean + SEM. Stimuli were compared using Student's *t*-test corrected for multiple comparisons using the Holm-Sidak method.

(B) J7774A.1 cells were pre-treated with SC514 (48 µM) and stimulated with AF SC (MOI=1), Zymosan (50 ug/ml) or LPS (25 ng/ml) for 6 hours. TNF-α was measured in the SN by ELISA. Multiple comparisons were performed after a two-way ANOVA.

(C) TNF-α mRNA levels were quantified by qPCR after a 6 hour stimulation with AF SC (MOI=5). SC514 significantly impaired the TNF-α response to AF. Bars show mean + SEM. Data was compared using Student's *t*-test.

Supplementary Figure 7: The AF-containing phagosome matures rapidly.

J774A.1 macrophages were infected with eGFP-expressing swollen conidia (MOI=3) and recruitment of (A) Rab5 and Rab7 and (B) acidification was assessed by confocal microscopy. Representative images of the indicated time points are shown at (A) 40x and (B) 60x magnification. Arrows indicate areas of recruitment to fungal conidia. All scale bars are 20 µm. (C) shows mean +/- SEM of the average pixel intensity for the indicated marker localizing to the AF-containing phagosome. Average pixel intensities were calculated using ImageJ.

Supplementary Figure 8: TLR9 and BTK are not required for *A. fumigatus* phagocytosis in macrophages.

J774A.1 macrophages were pre-treated with (A) ODN2088 (10 uM), (B) BTK inhibitor (12.5 uM) and (C) control or BTK targeting siRNA (25 nM). Cells were co-incubated with SC for 30 min and uptake was quantified by counting conidia positive cells. Bars represent mean+SEM. Statistical analysis was performed using Student's *t*-test.

Supplementary Figures and Tables

Supplementary Figure 1

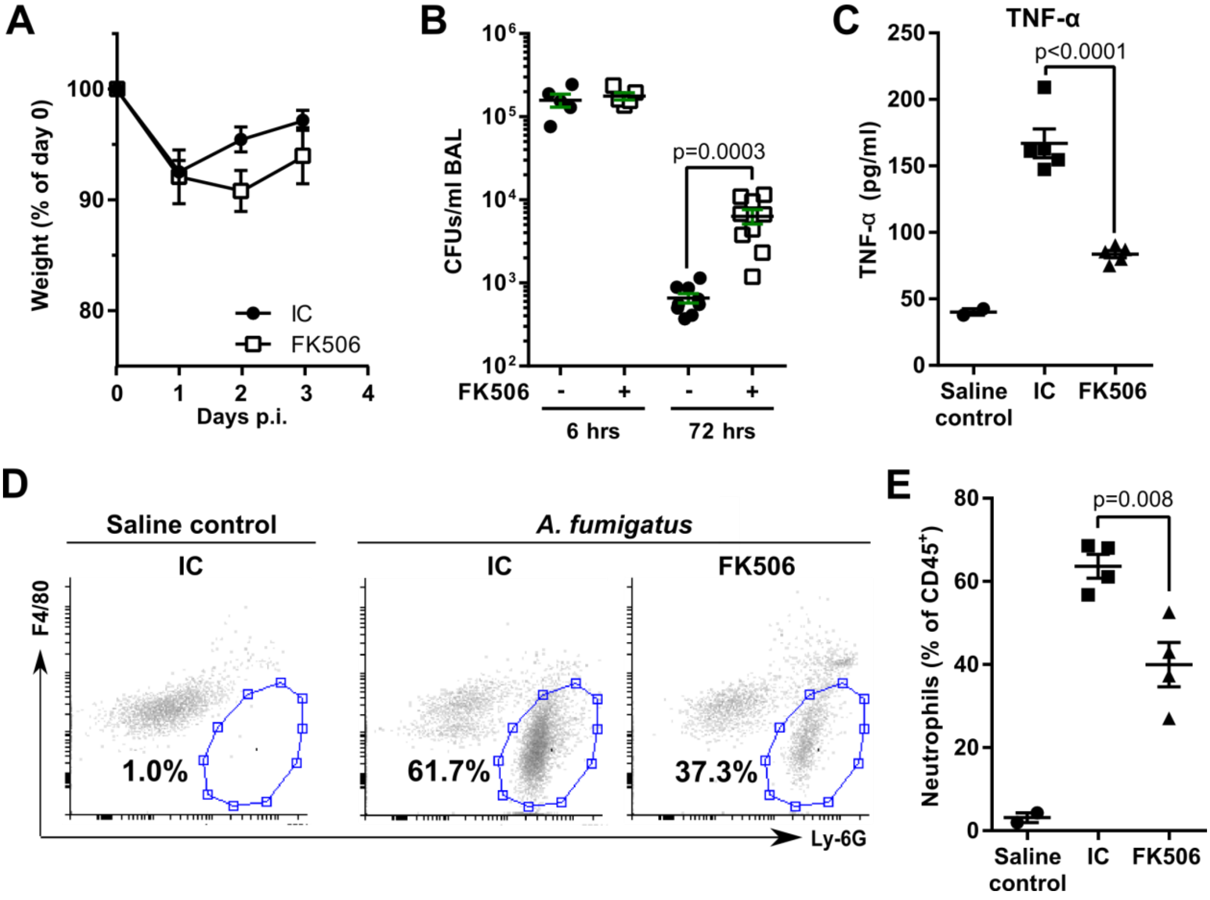

Supplementary Table 1

Protein	Gene	Amino acid identity to *Homo sapiens*
Calcineurin, catalytic subunit A	Protein phosphatase, catalytic subunit, α-isozyme (PPP3CA)	87 %
	Protein phosphatase, catalytic subunit, β-isozyme (PPP3CB)	88 %
	Protein phosphatase, catalytic subunit, γ-isozyme (PPP3CC)	81 %
Calcineurin, regulatory subunit B	Protein phosphatase, regulatory subunit B, α-isozyme (PPP3R1)	99 %
	Protein phosphatase, regulatory subunit B, α-isozyme (PPP3R2)	85 %
FK506-binding protein 12	FK506-binding protein 1Ab (FKBP1ab)	82 %

Supplementary Figure 2

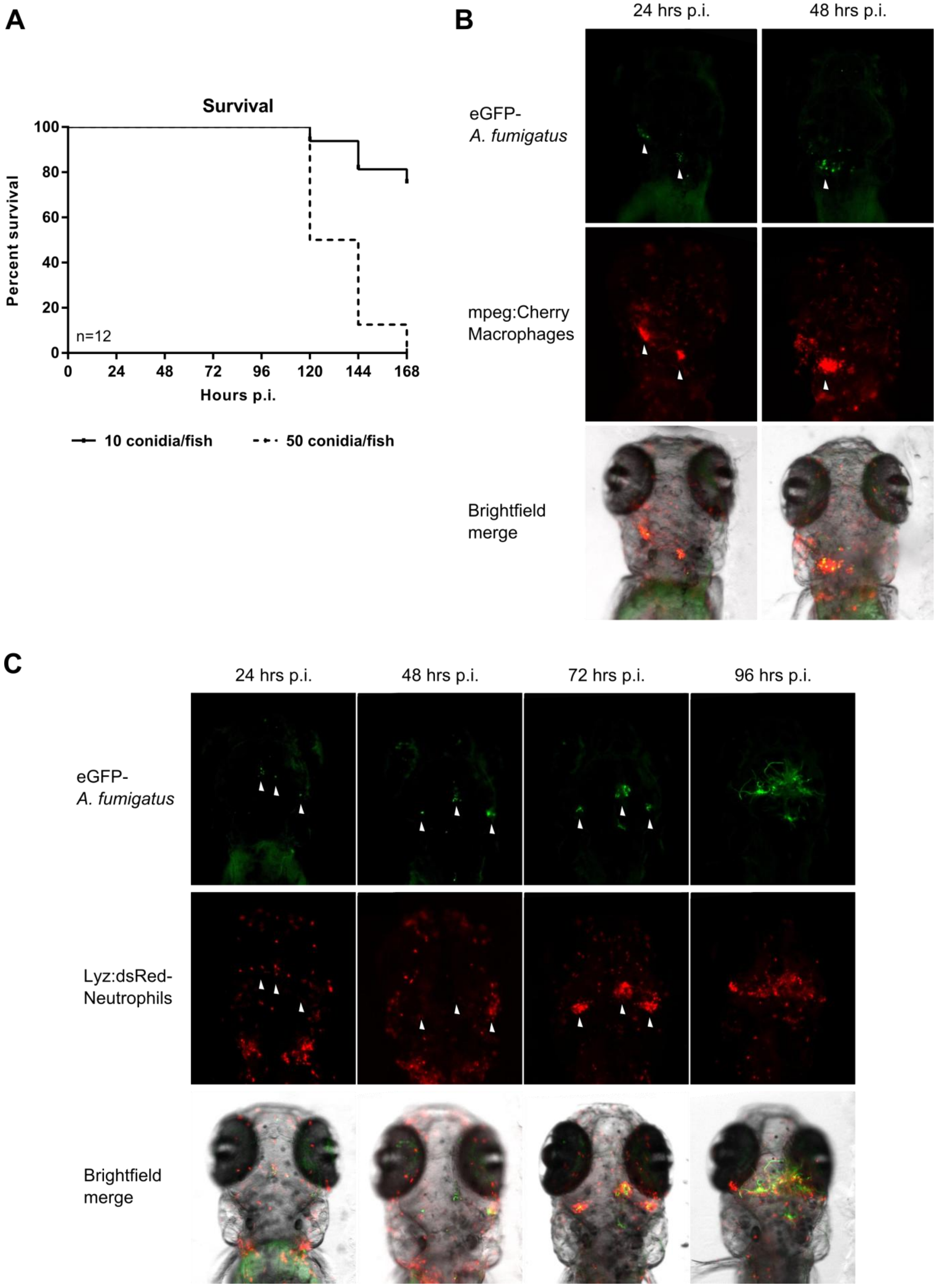

Supplementary Figure 3

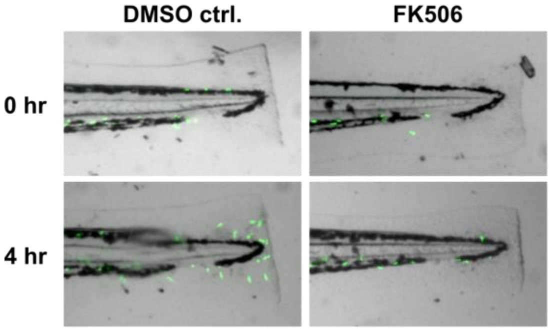

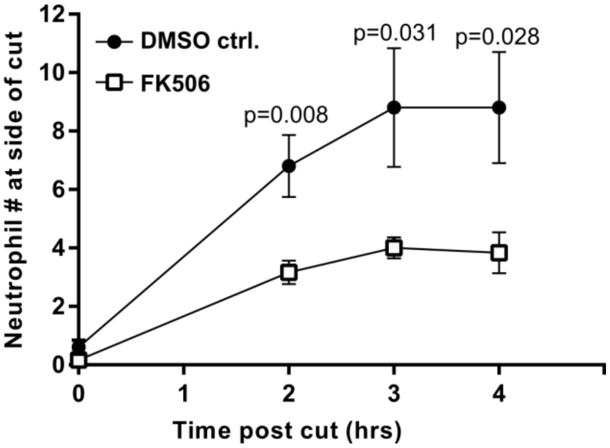

Supplementary Figure 4

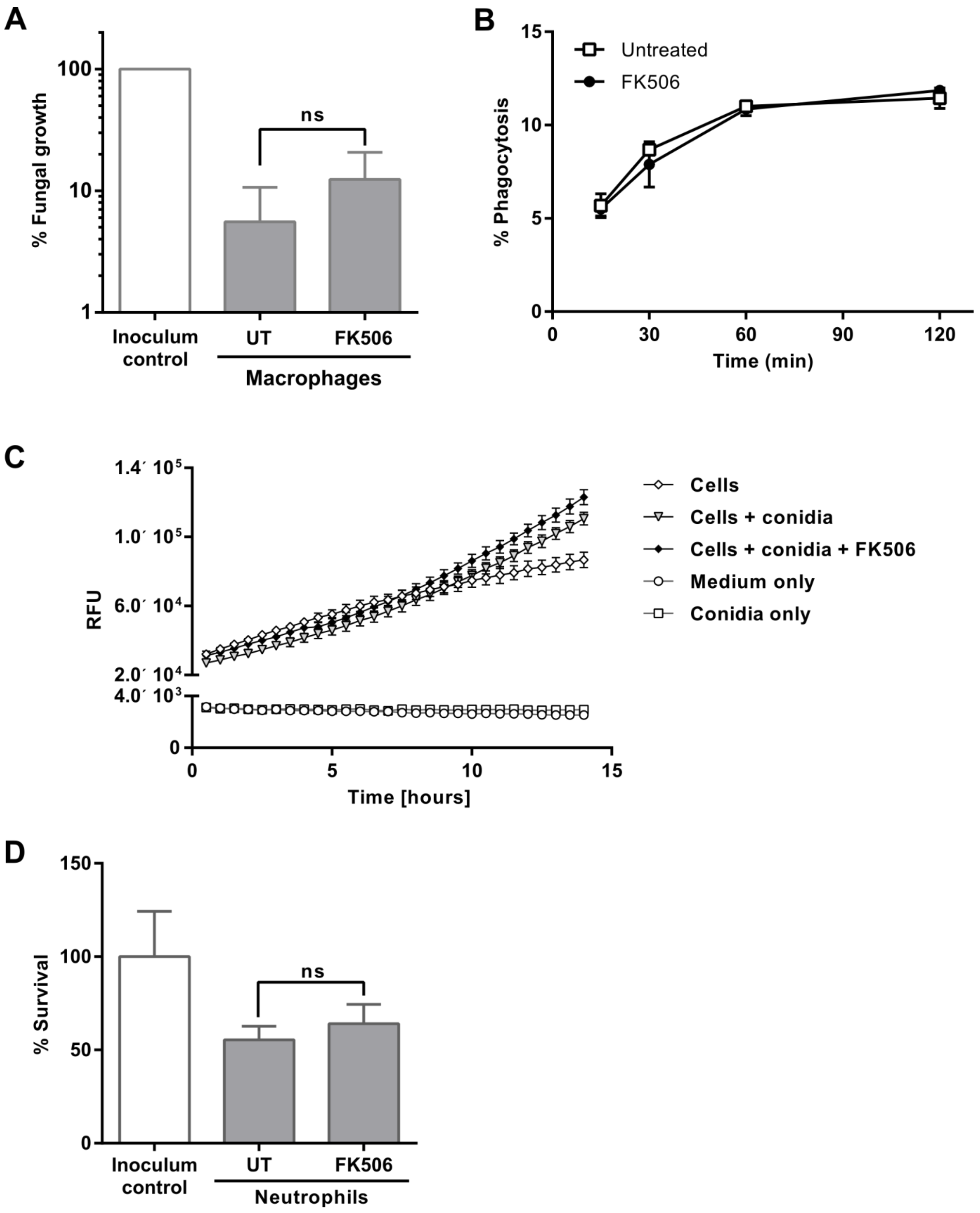

Supplementary Figure 5

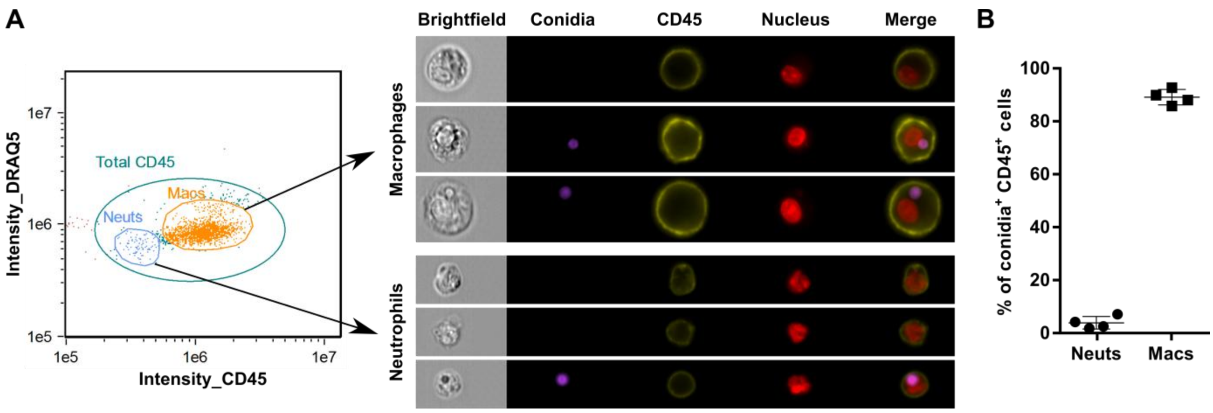

Supplementary Figure 6

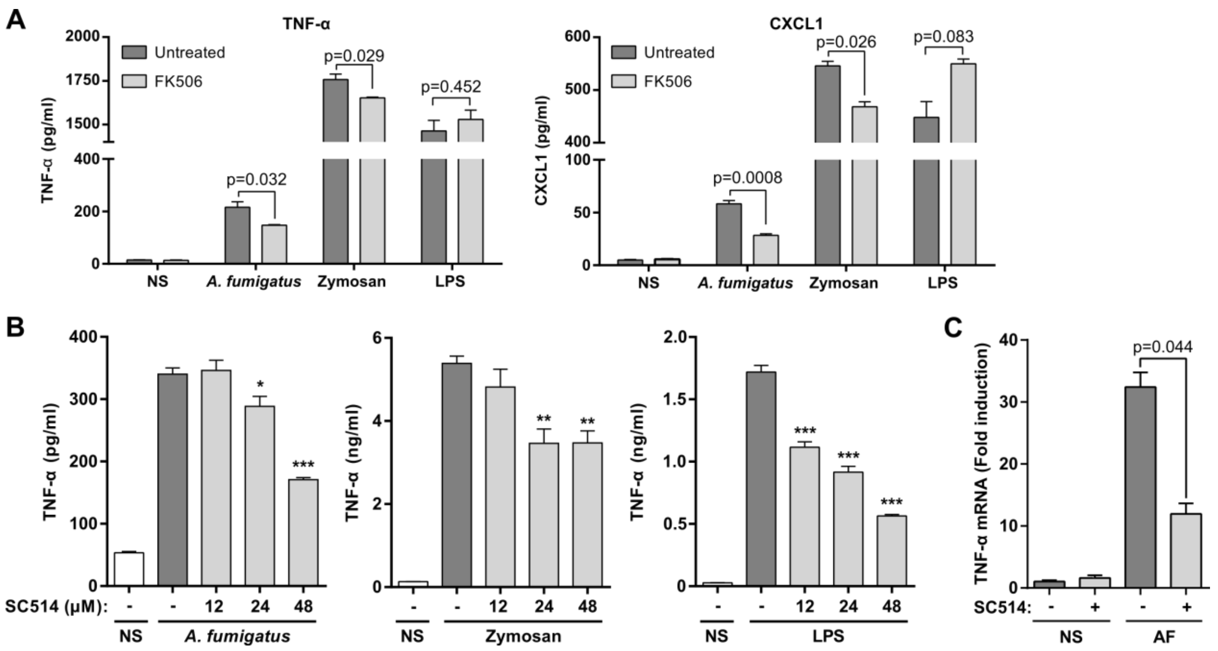

Supplementary Figure 7

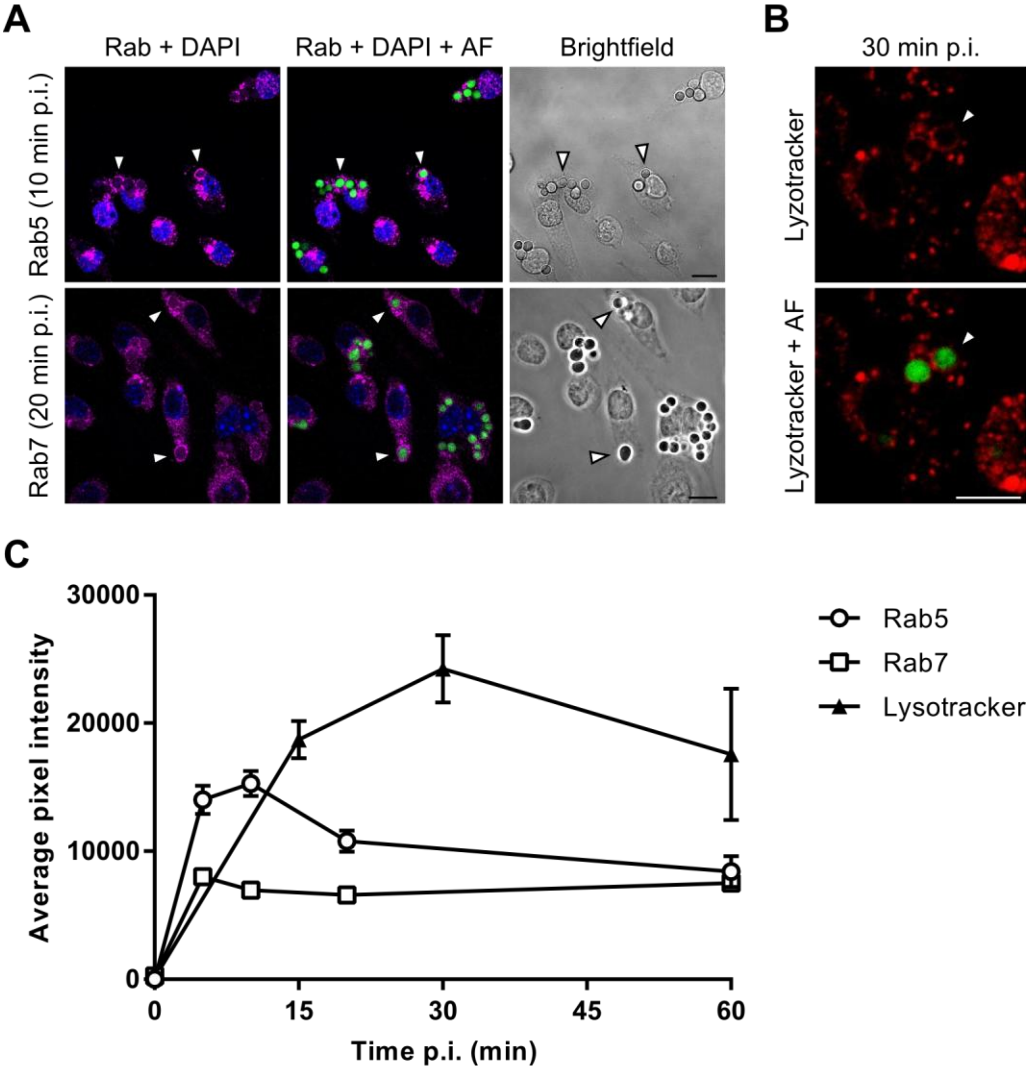

Supplementary Figure 8

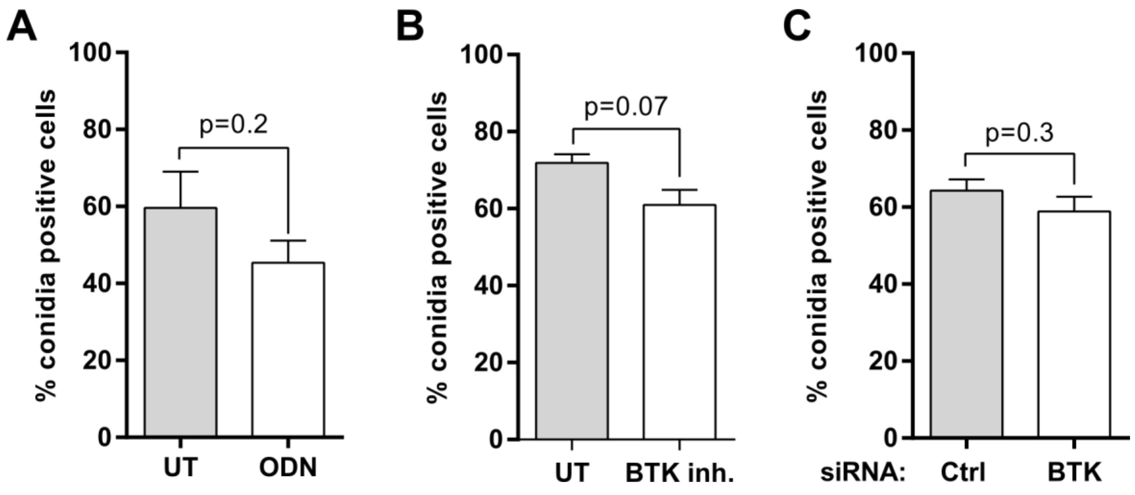

Supplementary Materials and Methods

Antibodies used in this study

Table 1 Antibodies used for Western Blotting and Confocal Microscopy

Antibody	Catalogue number	Dilution
anti-Glucocorticoid receptor, clone D8H2	3660, Cell Signaling	IF: 1:100
Anti-NFATc32, clone D43B1	5861, Cell Signaling	WB: 1:1000<br>IF: 1:20
anti-Rab5, clone C8B1	3547, Cell Signaling	IF: 1:50
anti-Rab7, clone D95F2	9367, Cell Signaling	IF: 1:50
anti-TLR9, polyclonal	PA5_20203, Thermo Scientific	WB: 1:500<br>IF: 1:50
anti-NFkB p65, clone C22B4	4764, Cell Signaling	WB: 1:2000
anti-HDAC1, clone 10E2	5356, Cell Signaling	WB: 1:2000
anti-Pan-calcineurin A, polyclonal	2614, Cell Signaling	WB: 1:100
anti-Syk, clone D3Z1E	13198, Cell Signaling	WB: 1:1000
anti-BTK, clone D3H5	8547, Cell Signaling	WB: 1:1000
anti-β-actin, clone 8H10D10	3700, Cell Signaling	WB: 1:2000

WB: Western Blotting; IF: Immunofluorescence

Table 2 Antibodies used for murine FACS analysis

Antibody	Catalogue number	Volume used per test
anti-F4/80-APC/Cy7, clone BM8	123118, Biolegend	2.5 µl
anti-Ly-6G-BV421, clone 1A8	127628, Biolegend	2.5 µl
anti-CD45- PE/Cy7, clone 30-F11	24-0451, eBioscience	1.5 µl
anti-CD45-PE, clone 30-F11	12-0451-82, eBioscience	1.5 µl
anti-CD11b-PE-CF594, clone M1/70	562317, BD	1 µl

Table 3 Antibodies used for human FACS analysis

Antibody	Catalogue number	Volume used per test
anti-CD11b-PerCP/Cy5.5, clone ICRF44	301327, Biolegend	3 µl
anti-CD11c-PE/Cy7, clone 3.9	301607, Biolegend	1 µl

anti-HLA-DR-APC/Cy7, clone L243	307617, Biolegend	1 µl
anti-CD206-Alexa647, clone 15-2	321116, Biolegend	1 µl
anti-CD86-BV421, clone IT2.2	305425, Biolegend	1.5 µl
