# Supplementary material for: Phagocytosis-dependent activation of a TLR9–BTK–calcineurin–NFAT pathway co-ordinates innate immunity to Aspergillus fumigatus
Source: EMBO Mol Med. 2015 Jan 30;7(3):240–58. doi: 10.15252/emmm.201404556 (PMC4364943; doi:10.15252/emmm.201404556)
Supplement: Supplementary file 2 [file emmm0007-0240-sd2.pdf]

EMBO
Molecular Medicine

# Phagocytosis-dependent activation of a TLR9-BTK-calcineurin-NFAT pathway coordinates innate immunity to *Aspergillus fumigatus*

Susanne Herbst, Anand Shah, Maria Mazon Moya, Vanessa Marzola, Barbara Jensen, Anna Reed, Mark A Birrell, Shinobu Saijo, Serge Mostowy, Sunil Shaunak and Darius Armstrong-James

*Corresponding author: Darius Armstrong-James, Imperial College London*

---

**Review timeline:**

| | |
|---|---|
| Submission date: | 18 August 2014 |
| Editorial Decision: | 24 September 2014 |
| Revision received: | 27 November 2014 |
| Editorial Decision: | 10 December 2014 |
| Revision received: | 29 December 2014 |
| Accepted: | 08 January 2015 |

---

## Transaction Report:

(Note: With the exception of the correction of typographical or spelling errors that could be a source of ambiguity, letters and reports are not edited. The original formatting of letters and referee reports may not be reflected in this compilation.)

*Editor: Céline Carret*

1st Editorial Decision 24 September 2014

Thank you for the submission of your manuscript to EMBO Molecular Medicine. Unfortunately, a referee dropped out at the last moment so we had to ask an editorial expert adviser to help reach the best possible decision. We have now heard back from the two referees whom we asked to evaluate your manuscript and from this advisor.

You will see that while Ref.1 raised serious concerns not supporting publication, Ref.3 found the study interesting and was much more positive about it, enthusiasm that was shared by our advisor who stated "I find the use of two models to show the role of calcineurin pathway a strength, rather than a weakness, as they complementary contribute to strengthen the argument. In addition, this is an important novel immunological pathway described for the immune response to *Aspergillus* (and other fungi). [...] reviewer 1 has some valid suggestions, that can be addressed".

Therefore, as we find the suggestions from both referees constructive and self explanatory enough, we would encourage you to address these criticisms in full and experimentally when requested. Should you be able to respond to all concerns satisfactorily, we would be happy to consider a revised manuscript.

Please note that it is EMBO Molecular Medicine policy to allow a single round of revision in order to avoid the delayed publication of research findings. Consequently, acceptance or rejection of the manuscript will depend on the completeness of your responses included in the next version of the manuscript.

© EMBO

EMBO Molecular Medicine has a "scooping protection" policy, whereby similar findings that are published by others during review or revision are not a criterion for rejection. Should you decide to submit a revised version, I do ask that you get in touch after three months if you have not completed it, to update us on the status.

Please also contact us as soon as possible if similar work is published elsewhere. If other work is published we may not be able to extend the revision period beyond three months.

I look forward to receiving your revised manuscript.

Should you find that the requested revisions are not feasible within the constraints outlined here and choose, therefore, to submit your paper elsewhere, we would welcome a message to this effect.

***** Reviewer's comments *****

Referee #1 (Comments on Novelty/Model System):

I think the use of the two unrelated models is a major weakeness of this work that overall does not convincingly provide new information regarding the role of Calcineurin/NFAT in aspergillosis.

Referee #1 (Remarks):

The authors reported that the calcineurin inhibitor tacrolimus impairs clearance of *Aspergillus* fumigatus from the airways of infected mice. They also resorted to a model of aspergillosis in zebrafish to confirm the murine data. They described a role for NFAT in the phagocytosis of either zymosan particle or *Aspergillus* conidia, described the signal transduction divergences and confirmed the dependence of TNF- on NFAT pathway in primary alveolar macrophages.
The work is of potential interest. However, there are major weaknesses of either conceptual or methodological nature.

Major points
1. In Figure 1 the analysis of Rag2-/- mice is incomplete. Please, provide the appropriate and required controls.
2. The observations made in vivo regarding neutrophils recruitment impairment following the FK506 treatment are not apparently related with the rest of the in vitro experiments done on macrophages.
3. While different cytokines are modulated, it is not clear the author's choice to focus on TNF- .
4. The data on the zebrafish model only partially overlaps with those obtained in mice. Thus, the authors need to clearly state in the paper why they resorted to the different models and the relative contribution of either one to the message of the paper. If not clearly positioned within the paper, the inclusion of the two models is perceived as being without a scientific rationale. As such, the two models are totally unrelated since the zebrafish model is an intra-brain infection while the mouse model is an intranasal infection.
5. The usage of FK506 does not completely prove the inhibition of Calcineurin/NFAT in innate immune cells such as macrophages and neutrophils, due to the wide expression of this pathway in different immune compartments. This may require the use of conditional knockout mice.

Referee #3 (Remarks):

The article by Herbst et al. describes a novel pathway in *Aspergillus* host defense. TLR9-BTK-NFAT pathway is involved in the production of TNFa by macrophages, which is inhibited by a calcineurin inhibitor FK506 (sirolimus) that is used in clinical practice. The decreased TNFa production, decreased chemokine production, and reduced neutrophil influx caused by FK506 as shown in the experimental models are convincing and have important clinical relevance. The data

are potentially very interesting, however, some issues need to be addressed in order to make more solid conclusions and especially the role of TLR9 in this pathway, is not yet convincing.

Major comments:
1. Are there any effects of FK506 in RAG-/- mice? Obviously the authors need to perform this experiment to know whether the effects of FK506 have indeed a relevant dramatic impact on innate immunity such as deficient neutrophil recruitment. This will also answer the question how much of the effects of FK506 *in vivo* are due to the presence of hydrocortisone. If the effects are dependent on the presence of hydrocortisone it should be investigated whether FK506 influences the killing of *Aspergillus* in HC-treated macrophages and neutrophils.
2. The authors have shown previously that murine macrophages in the presence of FK506 cannot efficiently inhibit fungal growth. Why this discrepancy? The authors need to discuss this.
3. Given 1 and 2 the authors need to investigate whether FK506 has effects on *Aspergillus* growth itself. This would also be important to know to interpret the fungal growth in the zebrafish model. This needs to be discussed.
4. TLR9 function thus far has been shown to be completely Myd88 dependent. This has not been challenged yet, and thus far no evidence of a TLR9-Myd88 independent function has been described. However, the authors show that Myd88-/- cells did not have an effect on NFATc2 translocation, while TLR9-/- does have this effect. To strengthen there hypothesis that this pathway is Myd88 independent the authors need to perform blocking TLR9 in Myd88-/- cells and look at NFAT2c translocation.
5. The authors need to investigate the effects of the BTK inhibitor in the TLR9-/- cells, and the BTK siRNA with the TLR9 blocker, to know whether the effects of BTK blocking that they observe are indeed dependent on this TLR9 pathway.
6. Since the induction of NFAT translocation is much higher in the J774 macrophages, and the differences with inhibition via siRNA are much clearer then in primary knockout cells, the authors need to conform that siRNA for TLR9 would indeed also have an impact on translocation by *Aspergillus*.

Minor comments:
1. Page 10 in heading says Brutonnd.

1st Revision - authors' response 27 November 2014

Referee #1 (Comments on Novelty/Model System):

I think the use of the two unrelated models is a major weakeness of this work that overall does not convincingly provide new information regarding the role of Calcineurin/NFAT in aspergillosis.

***Reply:***
*The zebrafish is an established model for infection that enables direct visualisation of innate host-pathogen interactions. We employed this model to enable direct visualisation of neutrophil recruitment in a new calcineurin-inhibitor based model of aspergillosis. We observed a defect in neutrophil recruitment to the airways in response to* Aspergillus *infection in our murine model of calcineurin-inhibition. We observed the same defect in neutrophil recruitment in our zebrafish model of calcineurin inhibition. The zebrafish model further allowed us to show that in those fungal foci where neutrophil recruitment did not occur, there was subsequent germination of fungal spores. Therefore the zebrafish model results supported the results from the murine model, and gave valuable extra direct visual confirmation of the requirement for neutrophil recruitment for control of fungal growth. Confirming the neutrophil recruitment defect in two different in vivo models strengthens the evidence for this crucial observation rather than weakening it. We have now further addressed this point on page 14 paragraph 3.*

Referee #1 (Remarks):

The authors reported that the calcineurin inhibitor tacrolimus impairs clearance of *Aspergillus fumigatus* from the airways of infected mice. They also resorted to a model of aspergillosis in zebrafish to confirm the murine data. They described a role for NFAT in the phagocytosis of either zymosan particle or *Aspergillus* conidia, described the signal transduction divergences and confirmed the dependence of TNF-a; on NFAT pathway in primary alveolar macrophages. The work is of potential interest. However, there are major weaknesses of either conceptual or methodological nature.

Major points

1. In Figure 1 the analysis of Rag2-/- mice is incomplete. Please, provide the appropriate and required controls.

***Reply:***

*We have now added the wild type control data from this experiment in Figure 1a (detailed on page 6 paragraph 1), which was not shown in the original manuscript submission. It demonstrates that a similar calcineurin-inhibitor dependent increase in mortality is seen when compared to the hydrocortisone-treated control animals. In addition, we now also show data confirming cytokine and neutrophil recruitment defects that are dependent upon calcineurin in Rag-/- mice, as requested by Reviewer 2 in Supplementary Figure 1 (detailed on page 7 paragraph 1).*

2. The observations made *in vivo* regarding neutrophils recruitment impairment following the FK506 treatment are not apparently related with the rest of the in vitro experiments done on macrophages.

***Reply:***

*Our observations indicate that defective calcineurin-dependent chemokine signalling in macrophages during the initial phagocytosis of* Aspergillus *leads to subsequent impaired neutrophil recruitment to the lung. This impaired neutrophil recruitment then leads to impaired host restriction of fungal growth. Therefore, whilst impaired neutrophil recruitment is the downstream defect that leads to a failure to control fungal growth efficiently, this is a consequence of an intrinsic defect of macrophage chemokine signalling in response to phagocytosis of* Aspergillus. *For this reason, we wished to further elucidate the mechanistic basis for this macrophage calcineurin-signalling defect. We have further clarified this in the discussion. We now also provide additional Imagestream data (Supplementary Figure 5 and page 8 paragraph 5) that demonstrates that at early time points post infection, and when the neutrophil defect is already apparent, 90% of the cells that have taken up conidia are macrophages. This observation further supports our finding that a defective early macrophage-dependent response is the key problem that leads to an increased fungal burden in the lung.*

3. While different cytokines are modulated, it is not clear the author's choice to focus on TNF-a.

***Reply:***

*Our initial in vivo data (Figure 2) identified multiple cytokines which were down-regulated by FK506 treatment (including TNF-α, IL-6, CXCL1, CXCL2 and CCL3). Of these cytokines, TNF-α has been shown to be important for fungal immunity, and it is also a key cytokine for neutrophil recruitment in murine models of pulmonary aspergillosis (Mehrad, B., Strieter, R. M. & Standiford, T. J. Immunol.* ***162,*** *1633–1640 (1999). Schelenz, S., Smith, D. A. & Bancroft, G. J. Med. Mycol.* ***37,***

*183–94 (1999). Furthermore, our clinical studies in transplant recipients have identified a key defect in TNF-α in transplant rceipients at high risk of life-threatening fungal disease (Armstrong-James D, et al. Am J Transplant. 2012 Dec;12(12):3437-40.). In addition, previous studies have shown that both NF-κB and NFAT bind to the TNF-α promoter (Macián, F., García-Rodríguez, C. & Rao, A. EMBO J.* ***19,*** *4783–95 (2000). For all of these reasons, we chose to focus on TNF-α as the key cytokine involved in antifungal immunity: it is regulated through known fungal response pathways via NF-κB, and also through the calcineurin pathway which we wished to explore further. This approach enabled us to continue dissecting the principle interactions and contributions of NF-κB and NFAT-dependent antifungal response pathways using a robust and reliable marker of innate fungal immunity. To further confirm the general relationship between calcineurin-signalling in macrophages and chemokine production, we have now added additional bone marrow-derived macrophage data on CXCL1 to supplement our data on TNF-α (Supplementary Data Figure 6a and page 8 paragraph 3). This is discussed further in page 14 paragraph 2 and page 16 paragraph 3.*

4. The data on the zebrafish model only partially overlaps with those obtained in mice. Thus, the authors need to clearly state in the paper why they resorted to the different models and the relative contribution of either one to the message of the paper. If not clearly positioned within the paper, the inclusion of the two models is perceived as being without a scientific rationale. As such, the two models are totally unrelated since the zebrafish model is an intra-brain infection while the mouse model is an intranasal infection.

***Reply:***

*We employed the zebrafish model as a tool for imaging the direct interaction between innate immune cells and* A. fumigatus *conidia. Whilst the mode of infection in zebrafish is necessarily different to the intranasal route, it is important to note that chemokine-based mechanisms of cell recruitment have been shown to be conserved in zebrafish (Sofia de Oliveira et al, J Immunol, 2013). We believe that our ability to demonstrate the same neutrophil defect in two different models is a strength of our paper rather than a weakness, because it shows a reproducible neutrophil recruitment defect in two different animal models with two different routes of infection. In addition, the ability to directly visualise host-pathogen interactions in the zebrafish model enabled further confirmation that when neutrophil recruitment to specific fungal foci was absent, clearance of fungal hyphae was impaired. This led to unchecked fungal growth and to death. We have now further addressed this point on page 14 paragraph 3.*

5. The usage of FK506 does not completely prove the inhibition of calcineurin/NFAT in innate immune cells such as macrophages and neutrophils, due to the wide expression of this pathway in different immune compartments. This may require the use of conditional knockout mice.

***Reply:***

*This is an interesting suggestion that we had also considered. However, unfortunately there is now increasing evidence that Cre-Lox systems are currently not able to discretely knock out genes in myeloid subsets (Hume, D; Applications of myeloid-specific promoters in transgenic mice support in vivo imaging and functional genomics but do not support the concept of distinct macrophage and dendritic cell lineages or roles in immunity. J. Leukoc. Biol.* ***89,*** *525–38 (2011). For this reason, we adopted an alternative strategy. First, we confirmed that there was indeed an innate calcineurin-based defect in innate fungal immunity using RAG-/- mice that lack adaptive immune responses. We then employed a combination of histopathological, FACS-based, iImgestream-based, cytokine-based, leukocyte-trafficking-based (i.e. zebrafish) and in vitro approaches to show that the initial defect in calcineurin-mediated innate immunity was in macrophages, and that it led to defective recruitment of neutrophils. Whilst it would be possible to further investigate these observations using Cre-Lox LysM mice, this would take 9-12 months to do (because of the requirement for cross-breeding). It could be the subject of future investigation and grant applications. However as we reason above, we do not think that the system would enable us to discriminate macrophage calcineurin responses from neutrophil calcineurin responses.*

Referee #3 (Remarks):

The article by Herbst et al. describes a novel pathway in *Aspergillus* host defense. TLR9-BTK-NFAT pathway is involved in the production of TNFa by macrophages, which is inhibited by a calcineurin inhibitor FK506 (sirolimus) that is used in clinical practice. The decreased TNF-a production, decreased chemokine production, and reduced neutrophil influx caused by FK506 as shown in the experimental models are convincing and have important clinical relevance. The data are potentially very interesting, however, some issues need to be addressed in order to make more solid conclusions and especially the role of TLR9 in this pathway, is not yet convincing.

Major comments:

1. Are there any effects of FK506 in RAG-/- mice? Obviously the authors need to perform this experiment to know whether the effects of FK506 have indeed a relevant dramatic impact on innate immunity such as deficient neutrophil recruitment. This will also answer the question how much of the effects of FK506 in vivo are due to the presence of hydrocortisone. If the effects are dependent on the presence of hydrocortisone it should be investigated whether FK506 influences the killing of *Aspergillus* in HC-treated macrophages and neutrophils.

***Reply:***

*We agree. As suggested, we have now repeated the calcineurin inhibition experiments in RAG-/- mice and found that our observations from wild-type mice are reproducible. These data are now included in Supplementary Figure 1 (detailed on page 7 paragraph 1). Calcineurin inhibition leads to defective fungal clearance, impaired TNF-α production and impaired neutrophil recruitment in RAG-/- mice. The effects are not dependent on the presence of hydrocortisone.*

2. The authors have shown previously that murine macrophages in the presence of FK506 cannot efficiently inhibit fungal growth. Why this discrepancy? The authors need to discuss this.

***Reply:***

*We previously found a defect in the control of fungal growth in MH-S cells, which are a BALBc alveolar macrophage-derived cell line that has been immortalised using SV40 constructs. However, subsequent experimentation has shown that MH-S cells have a poor calcineurin-NFAT response. This makes them a poor in vitro model.*

*In contrast to MH-S cells, FK506-treated BMDMs or J774A.1 cells did not show a defect in fungal growth inhibition. Because BMDMs are primary cells with higher antifungal activity and the macrophage cell line J774A.1 showed a comparable phenotype as measured by fungal killing, cytokine responses and calcineurin activation, we chose to undertake all further analysis in these (J774A.1) cells. As suggested we have now addressed this additional point in the manuscript discussion on page 15 paragraph 1.*

3. Given 1 and 2 the authors need to investigate whether FK506 has effects on *Aspergillus* growth itself. This would also be important to know to interpret the fungal growth in the zebrafish model. This needs to be discussed.

*We have previously demonstrated that FK506 has significant antifungal effects against* Aspergillus *at human-relevant therapeutic concentrations, and that these levels are achieved in our murine models (Herbst S, Shah A, Carby M, Chusney G, Kikkeri N, Dorling A, Bignell E, Shaunak S,* ***Armstrong-James D.*** *Dis Model Mech. May;6(3):643-51). Given these observations, our murine and zebrafish based observations indicate that the natural antifungal effects of FK506 are strongly*

*outweighed by its effects on innate immunity in vivo, leading to an overall increased risk of mortality from invasive fungal infection. We have now addressed this important point in the manuscript discussion on page 14 paragraph 3.*

4. TLR9 function thus far has been shown to be completely Myd88 dependent. This has not been challenged yet, and thus far no evidence of a TLR9-Myd88 independent function has been described. However, the authors show that Myd88-/- cells did not have an effect on NFATc2 translocation, while TLR9-/- does have this effect. To strengthen their hypothesis that this pathway is Myd88 independent the authors need to perform blocking TLR9 in Myd88-/- cells and look at NFAT2c translocation.

***Reply:***

*We have now performed these experiments. We found that blocking TLR9 signalling in Myd88 BMDMs did indeed lead to impaired NFAT responses to* Aspergillus fumigatus*, as now shown in Figure 7g and detailed on page 11 paragraph 2. This is also discussed on page 16 paragraph 1.*

*We are grateful for this excellent suggestion, which we believe strengthens the paper and further supports the finding that the pathway is Myd88-independent.*

5. The authors need to investigate the effects of the BTK inhibitor in the TLR9-/- cells, and the BTK siRNA with the TLR9 blocker, to know whether the effects of BTK blocking that they observe are indeed dependent on this TLR9 pathway.

***Reply:***

*We have now performed additional experiments to address these points. We find that inhibition of TLR9 in BTK knock-down macrophages (and vice versa) confers no additional inhibition of the NFAT response to* Aspergillus*, and as such is consistent with TLR9 and BTK acting through the same pathway. These data are now shown in Figure 8 D and E and detailed on page 11 paragraph 3. This is also discussed on page 16 paragraph 1.*

*We are grateful to the reviewer for these suggestions; we believe that the extra experiments significantly enhance the revised manuscript's conclusions.*

6. Since the induction of NFAT translocation is much higher in the J774 macrophages, and the differences with inhibition via siRNA are much clearer then in primary knockout cells, the authors need to conform that siRNA for TLR9 would indeed also have an impact on translocation by *Aspergillus*.

***Reply:***

*We have now addressed this as described in the previous section. TLR9 siRNA does indeed have significant impact on NFAT translocation, as shown in Figure 8 E and detailed on page 12 paragraph 1. This is also discussed on page 16 paragraph 1.*

Minor comments:

1. Page 10 in heading says Brutonnd.

***Reply:***

*This has now been corrected to Bruton's.*

2nd Editorial Decision 10 December 2014

Thank you for the submission of your revised manuscript to EMBO Molecular Medicine. We have now received the enclosed report from the referee asked to re-assess it. As you will see this reviewer is now fully supportive and I am pleased to inform you that we will be able to accept your manuscript pending final editorial amendments.

Please submit your revised manuscript within two weeks. I look forward to seeing a revised form of your manuscript as soon as possible.

***** Reviewer's comments *****

Referee #3 (Comments on Novelty/Model System):

The authors have adequately addressed all the issues raised and provide clinically relevant and novel findings.

Referee #3 (Remarks):

The authors have adequately addressed all the issues raised and provide clinically relevant and novel findings.
