# Supplementary material for: Phagocytosis-dependent activation of a TLR9–BTK–calcineurin–NFAT pathway co-ordinates innate immunity to Aspergillus fumigatus
Source: EMBO Mol Med. 2015 Jan 30;7(3):240–58. doi: 10.15252/emmm.201404556 (PMC4364943; doi:10.15252/emmm.201404556)
Supplement: Supplementary file 3 [file emmm0007-0240-sd3.pdf]

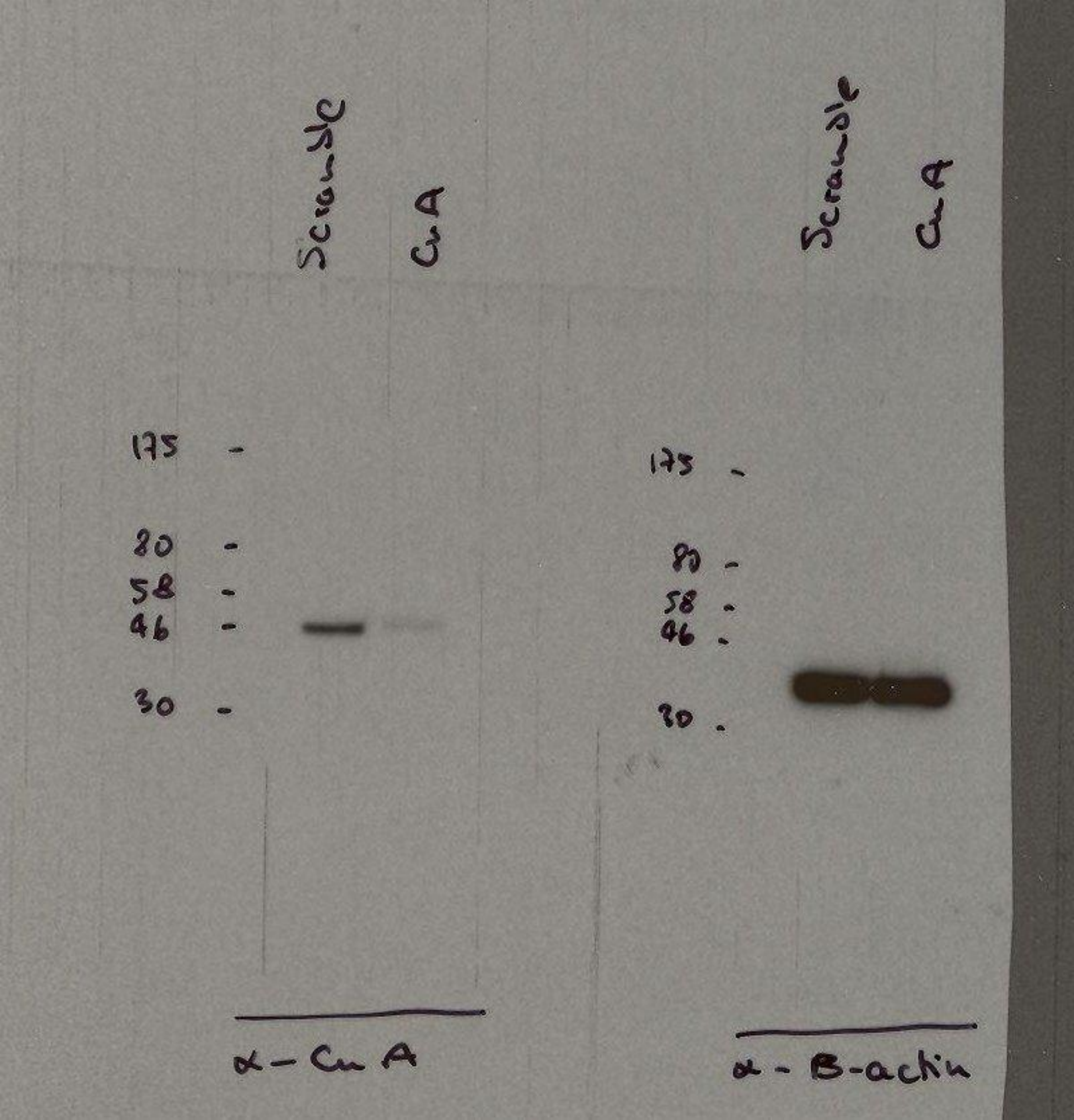
Scramble
CnA
175
80
58
46
30
α - CnA
Scramble
CnA
175
80
58
46
30
α - B-actin
