# Supplementary material for: Phagocytosis-dependent activation of a TLR9–BTK–calcineurin–NFAT pathway co-ordinates innate immunity to Aspergillus fumigatus
Source: EMBO Mol Med. 2015 Jan 30;7(3):240–58. doi: 10.15252/emmm.201404556 (PMC4364943; doi:10.15252/emmm.201404556)
Supplement: Supplementary file 4 [file emmm0007-0240-sd4.pdf]

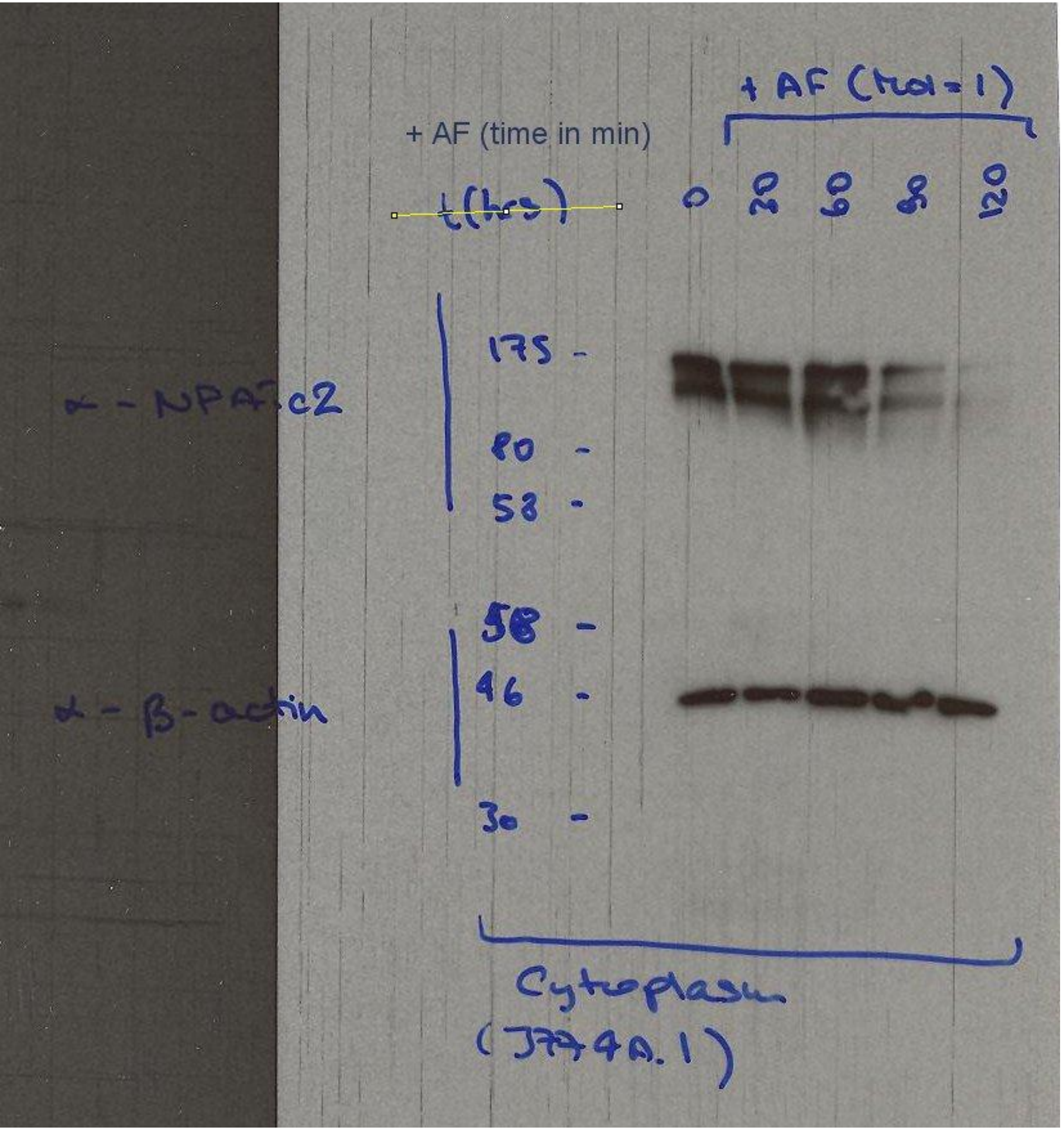
+ AF (time in min)
+ AF (MOI = 1)
0
30
60
90
120
α - NFATc2
175 -
80 -
58 -
58 -
46 -
α - β-actin
30 -
Cytoplasm
(J774A.1)

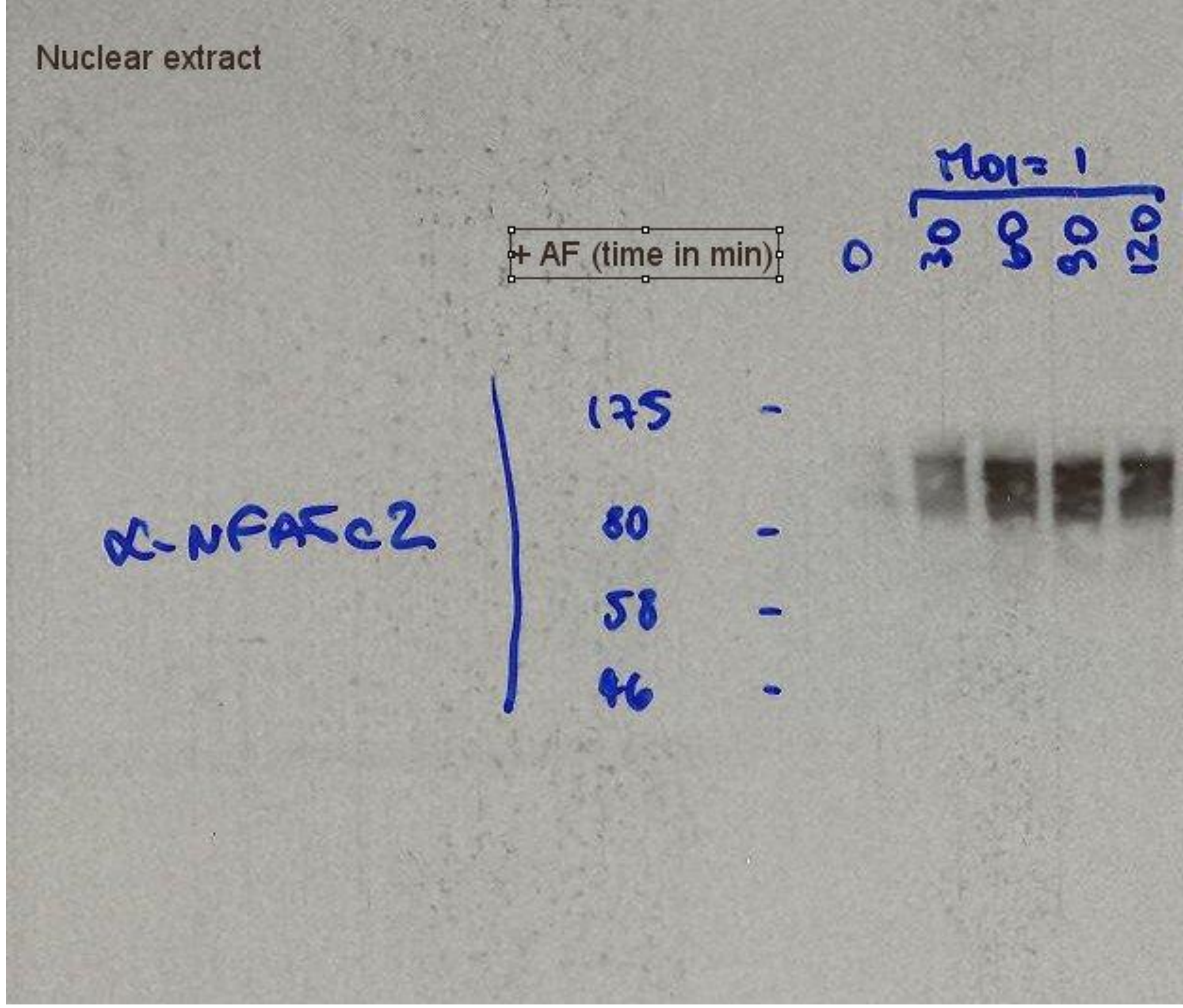
Nuclear extract
MOI = 1
+ AF (time in min)
0
30
60
90
120
α-NFATc2
175 -
80 -
58 -
46 -

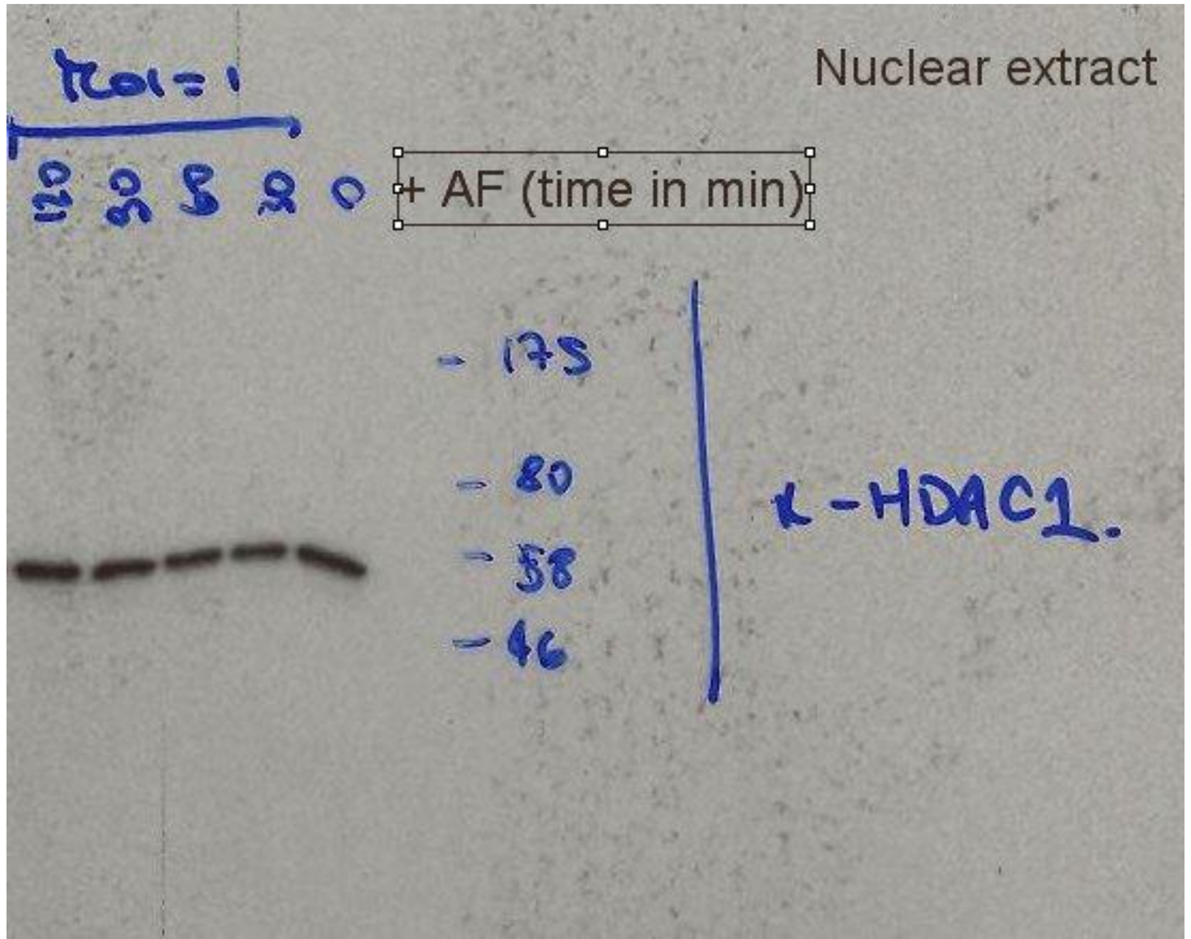
Nuclear extract
MOI = 1
120
90
60
30
0
+ AF (time in min)
- 175
- 80
- 58
- 46
α-HDAC1.

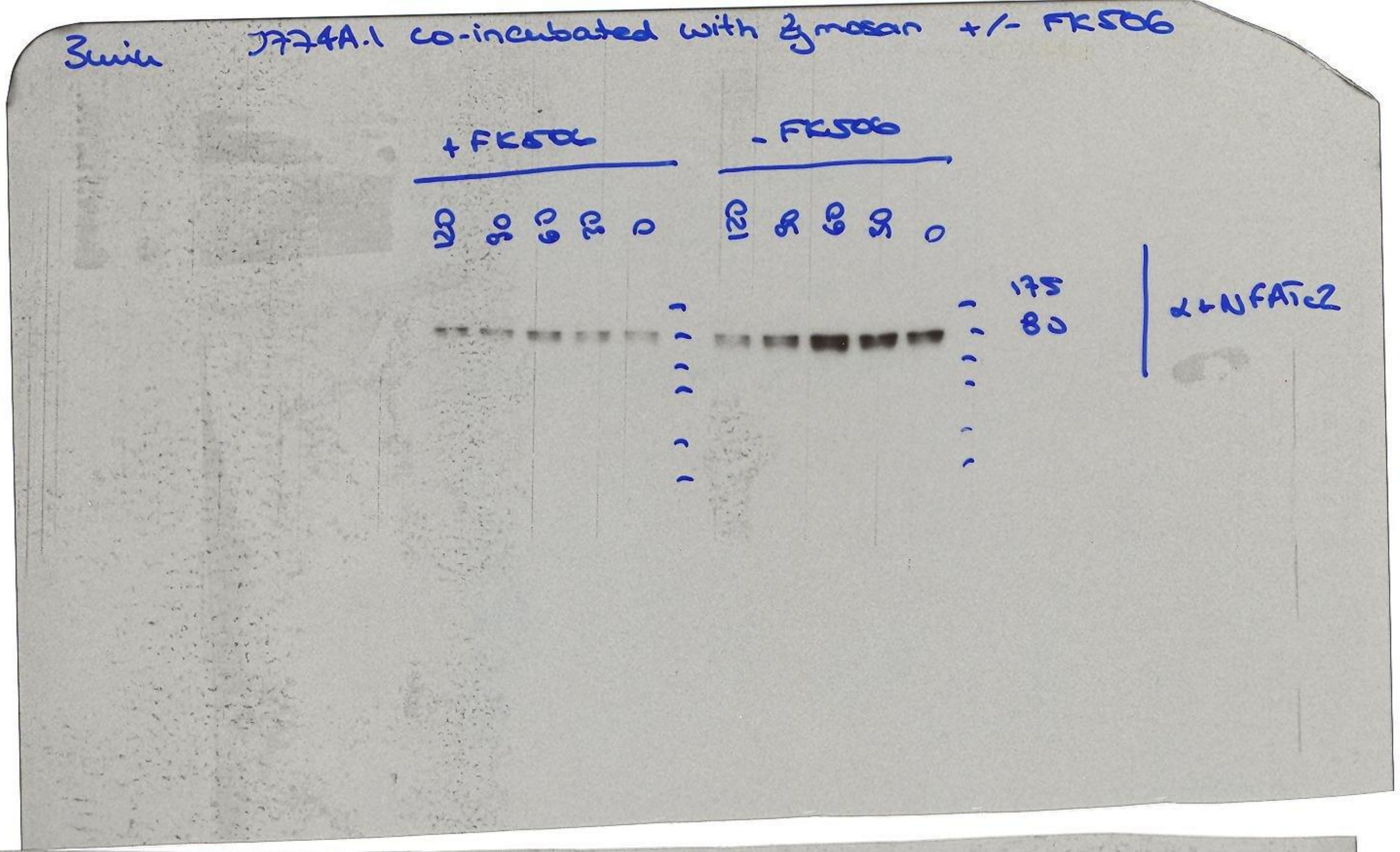
3min
J774A.1 co-incubated with Zymosan +/- FK506
+ FK506
- FK506
120 60 30 15 0
120 60 30 15 0
175
80
α-NFATc2

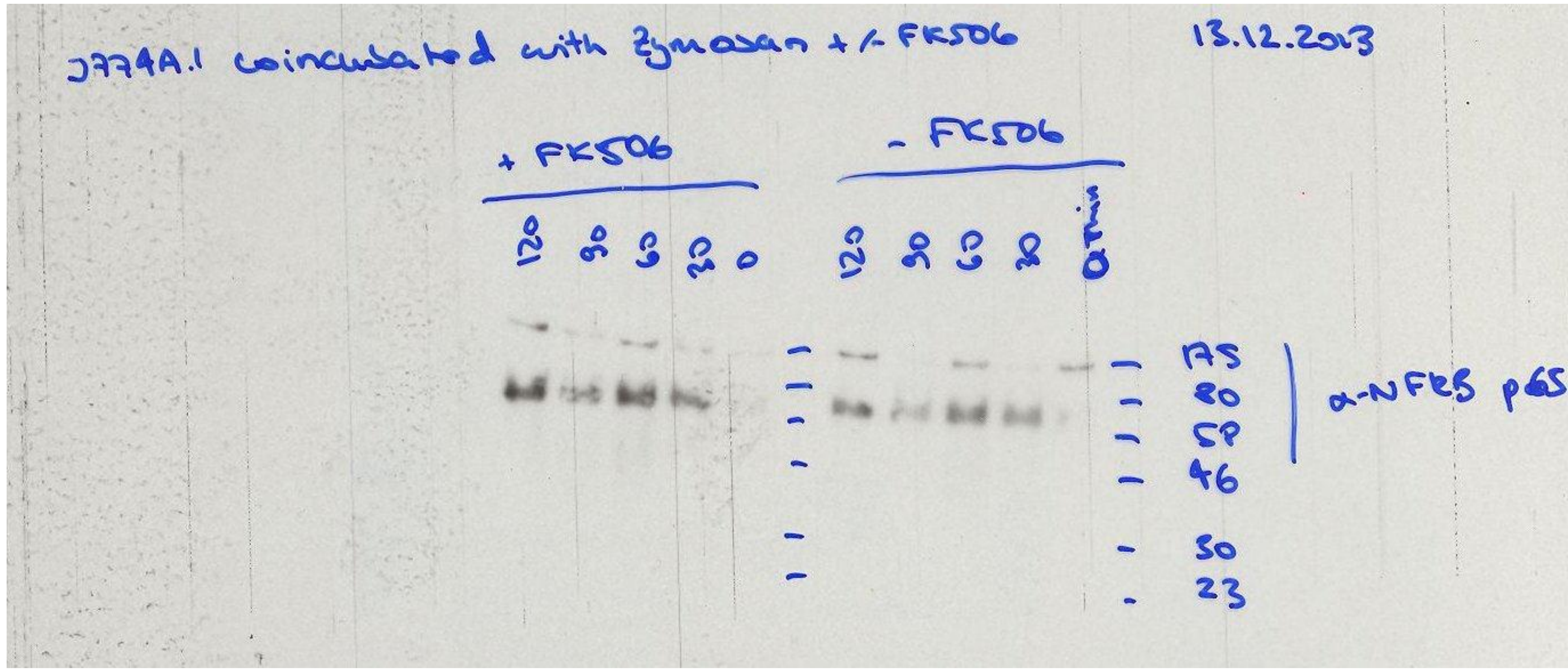
J774A.1 coincubated with Zymosan +/- FK506
13.12.2013
+ FK506
- FK506
120 60 30 15 0
120 60 30 15 0 min
175
80
58
46
30
23
α-NFκB p65

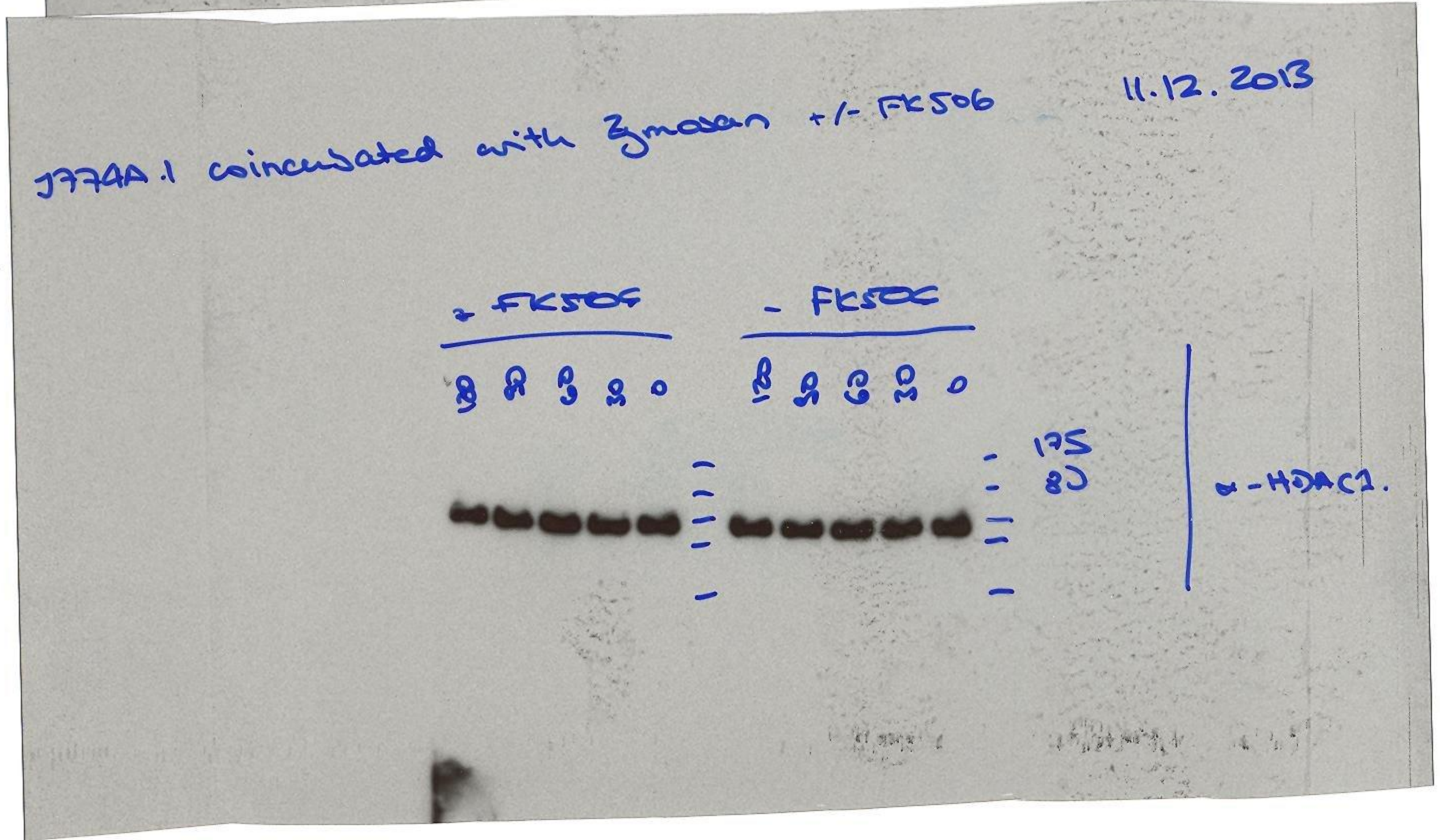
J774A.1 coincubated with Zymosan +/- FK506
11.12.2013
+ FK506
- FK506
120 60 30 15 0
120 60 30 15 0
175
80
α-HDAC1.
