# Supplementary figures and images for: Phagocytosis-dependent activation of a TLR9–BTK–calcineurin–NFAT pathway co-ordinates innate immunity to Aspergillus fumigatus
Source: EMBO Mol Med. 2015 Jan 30;7(3):240–58. doi: 10.15252/emmm.201404556 (PMC4364943; doi:10.15252/emmm.201404556)

$\alpha$ - $\beta$ -actin

19.04.2014

Ctrl. siRNA  
Eyk siRNA

80 -  
58 -  
46 -  
30 -

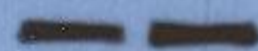

$\alpha$ -Syk

19.04.2014

Ctrl. siRNA  
Eyk siRNA

125 -  
80 -  
58 -  
46 -

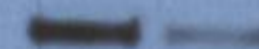

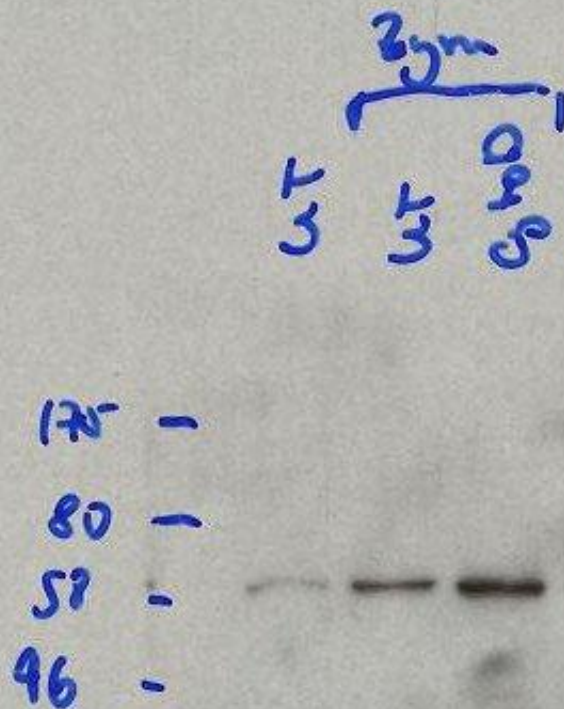

anti-NFkB p65

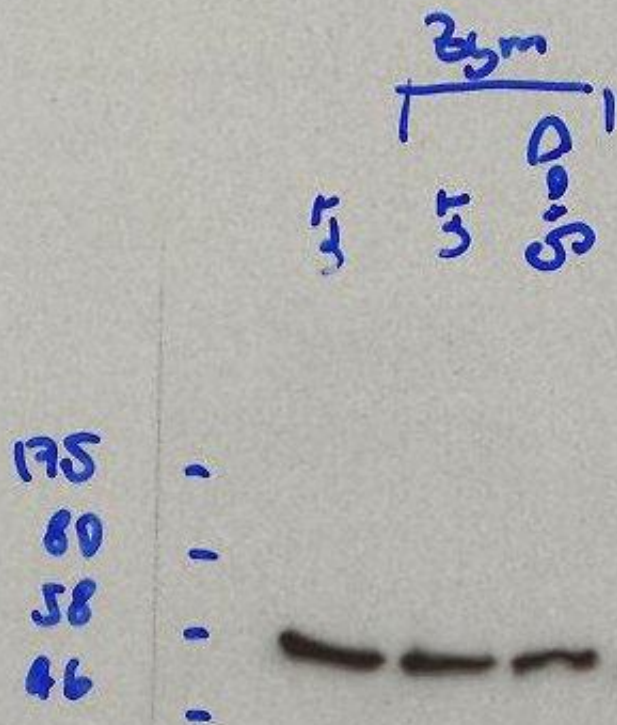

anti-HDAC1

Supplement: Supplementary file 5 [file emmm0007-0240-sd5.pdf]

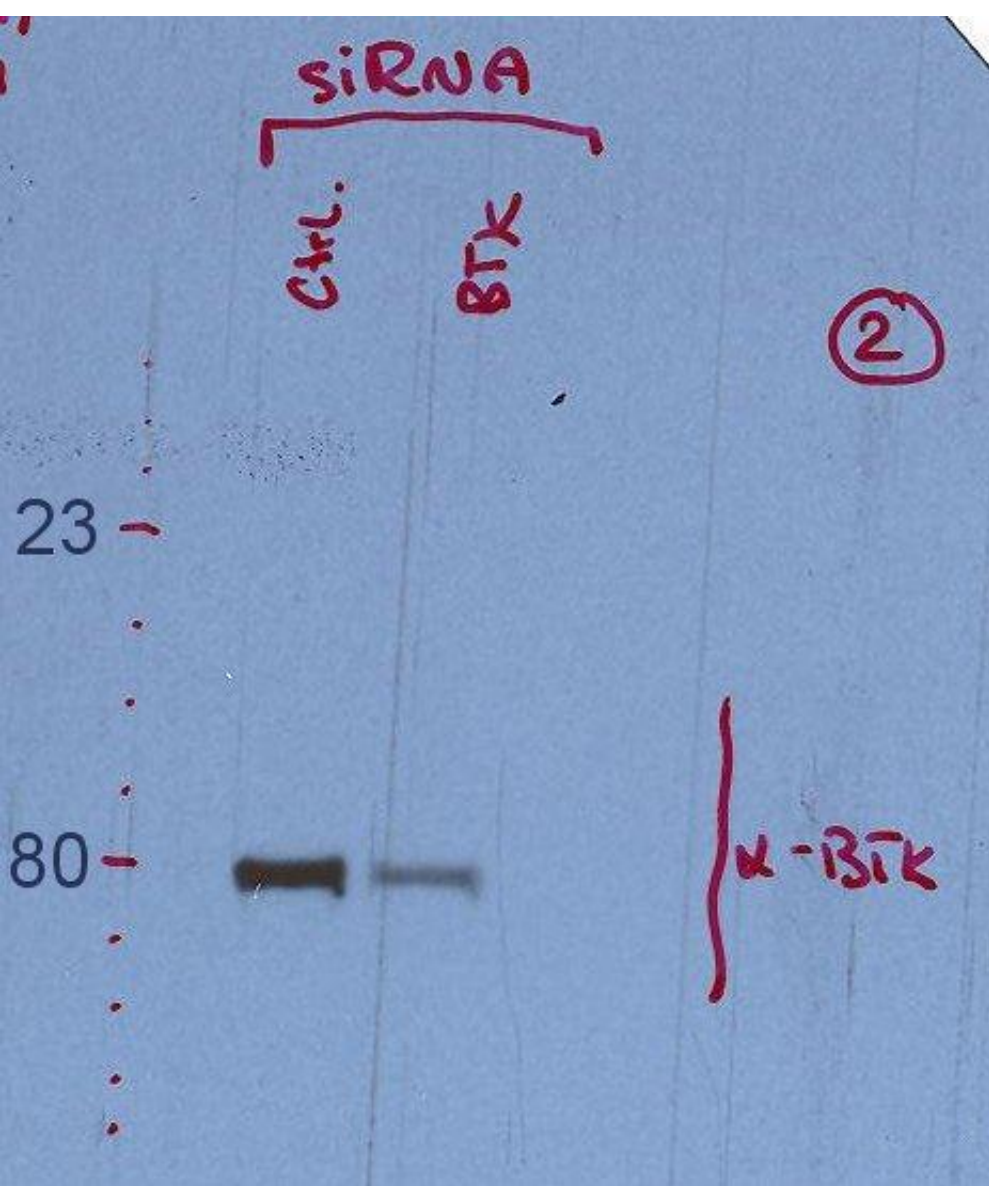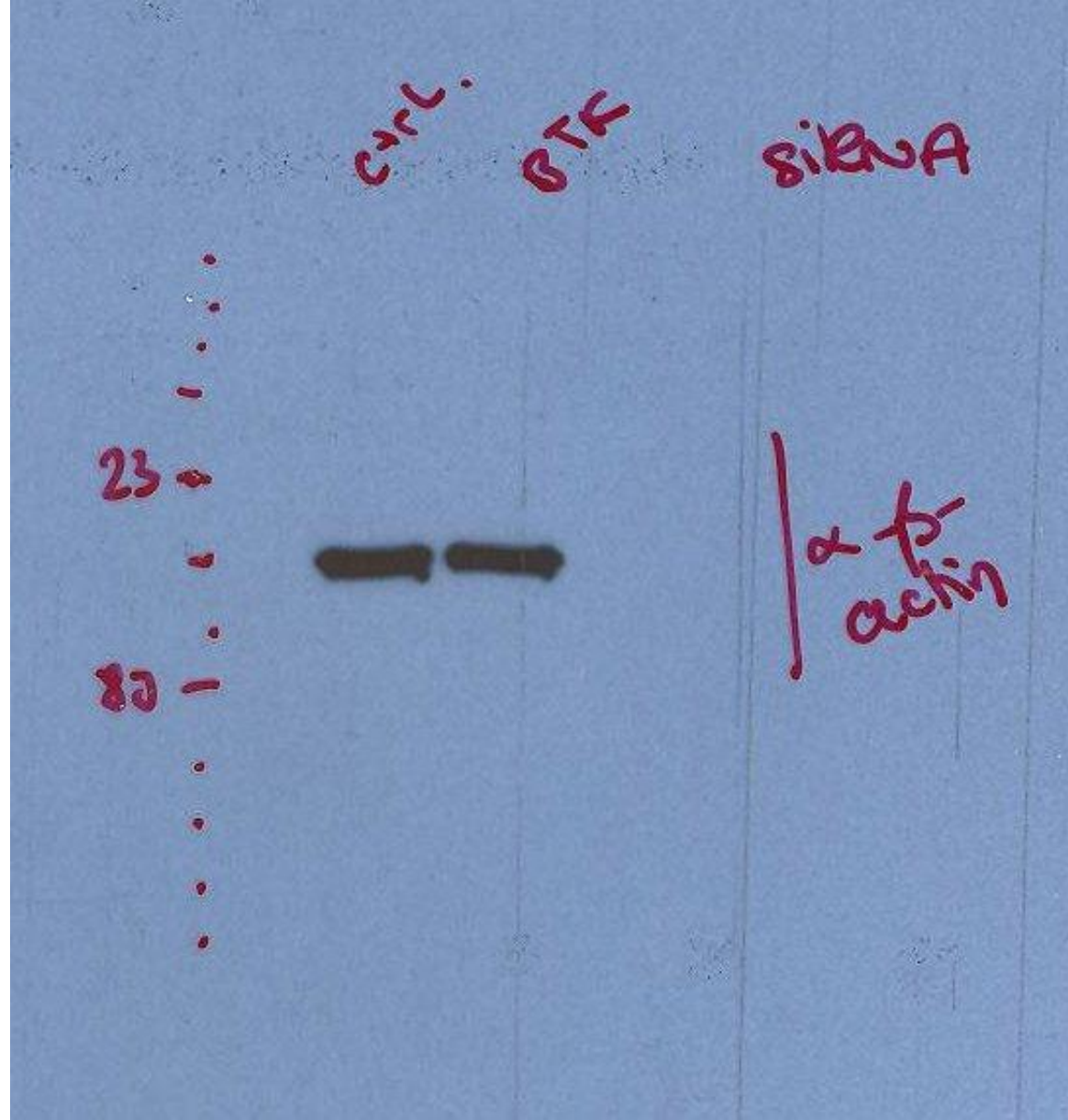

$\alpha$ -TUB

23  
30  
46  
58  
80  
100  
135

75nM  
25nM  
Ctrl. (75nM)  
TLR9 siRNA

$\beta$ -actin

23  
30  
46  
58  
80

75nM  
25nM  
Ctrl. (75nM)  
TLR9 siRNA

Supplement: Supplementary file 7 [file emmm0007-0240-sd7.pdf]
