# Supplementary material for: Phagocytosis-dependent activation of a TLR9–BTK–calcineurin–NFAT pathway co-ordinates innate immunity to Aspergillus fumigatus
Source: EMBO Mol Med. 2015 Jan 30;7(3):240–58. doi: 10.15252/emmm.201404556 (PMC4364943; doi:10.15252/emmm.201404556)
Supplement: Supplementary file 6 [file emmm0007-0240-sd6.pdf]

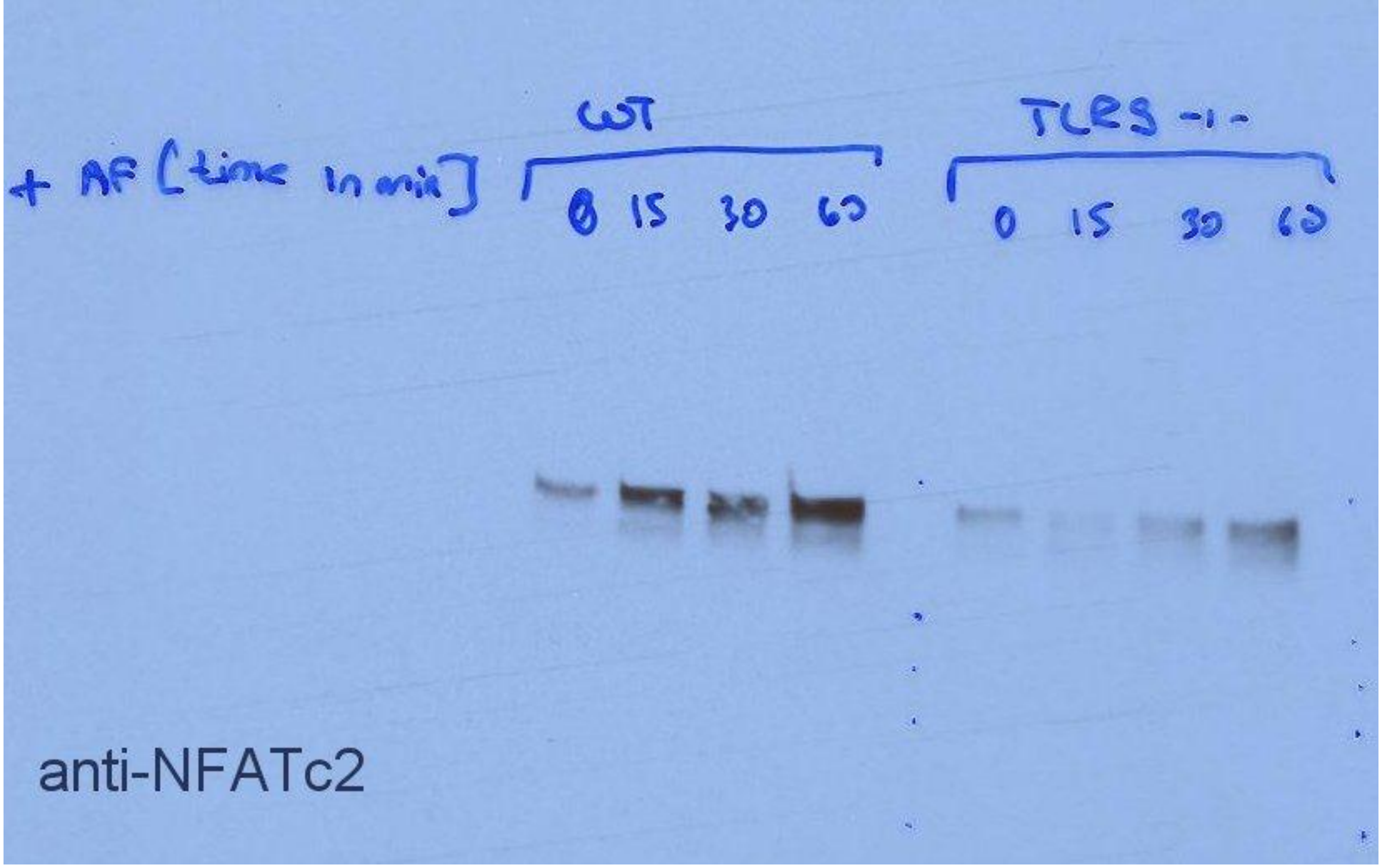
WT
TLR9 -/-
+ AF [time in min]
0 15 30 60
0 15 30 60
anti-NFATc2

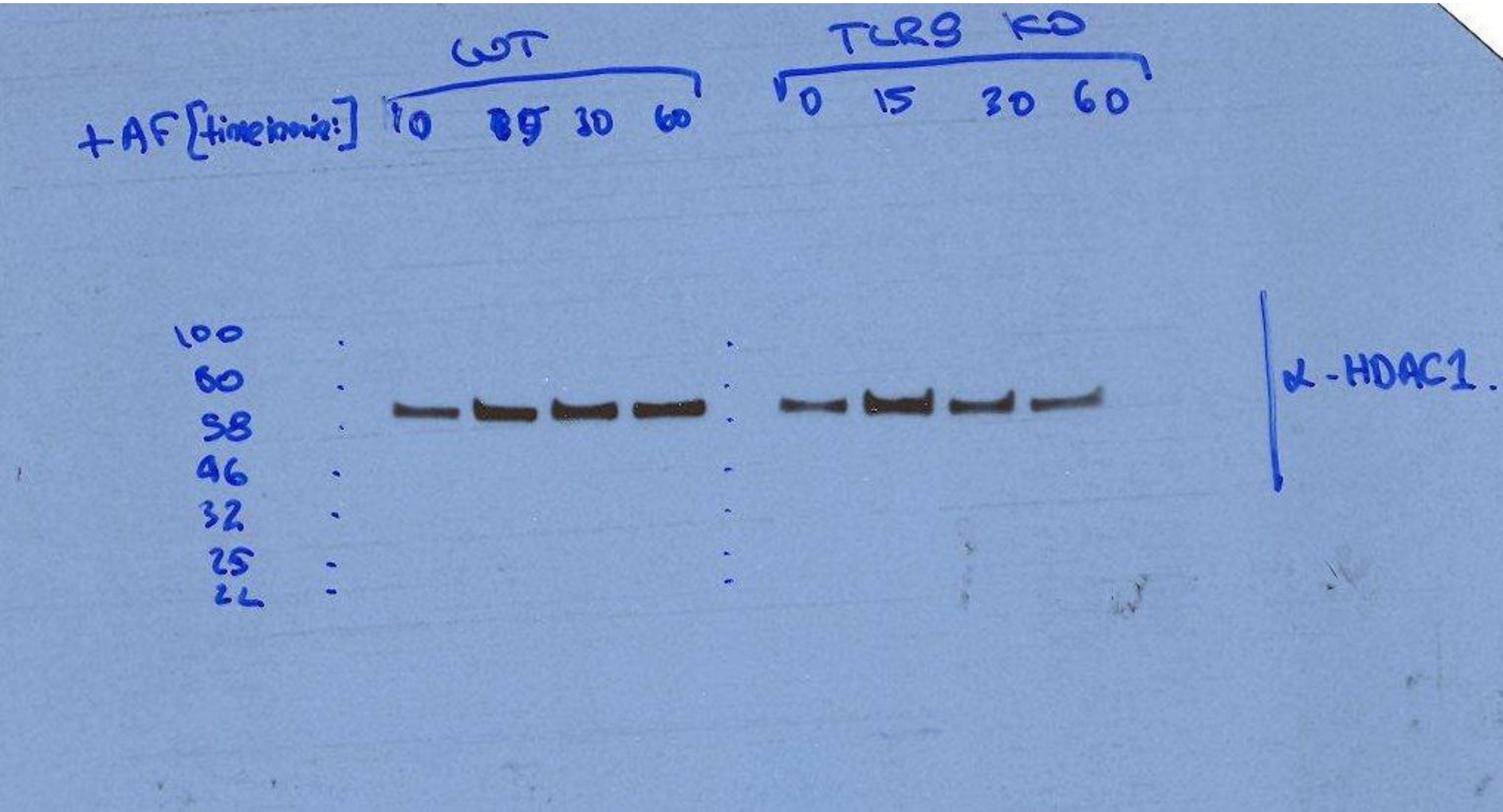
WT
TLR9 KO
+ AF [time in min]
0 15 30 60
0 15 30 60
100
80
58
46
32
25
22
α-HDAC1
